# Nuclear receptor modulators inhibit osteosarcoma cell proliferation and tumour growth by regulating the mTOR signaling pathway

**DOI:** 10.1038/s41419-022-05545-7

**Published:** 2023-01-21

**Authors:** Baoshi Yuan, Kexin Shi, Juanmin Zha, Yujia Cai, Yue Gu, Kai Huang, Wenchang Yue, Qiaocheng Zhai, Ning Ding, Wenyan Ren, Weiqi He, Ying Xu, Tao Wang

**Affiliations:** 1 Cambridge-Su Genomic Resource Center, Suzhou medical college of Soochow University, Suzhou, Jiangsu 215123 China; 2 Ministry of Education Key Laboratory of Biosystems Homeostasis and Protection and Innovation Center for Cell Signaling Network, Life Sciences Institute, Zhejiang University, Hangzhou, Zhejiang 310030 China; 3 Department of Oncology, The First Affiliated Hospital of Soochow University, Suzhou, Jiangsu 215006 China; 4 Department of Urology, The First Affiliated Hospital of Soochow University, Suzhou, Jiangsu 215006 China; 5 Department of Orthopaedics, the Second Affiliated Hospital of Soochow University, Suzhou, Jiangsu 215004 China

**Keywords:** Cancer therapy, Sarcoma

## Abstract

Osteosarcoma is the most common primary malignant bone tumour in children and adolescents. Chemoresistance leads to poor responses to conventional therapy in patients with osteosarcoma. The discovery of novel effective therapeutic targets and drugs is still the main focus of osteosarcoma research. Nuclear receptors (NRs) have shown substantial promise as novel therapeutic targets for various cancers. In the present study, we performed a drug screen using 29 chemicals that specifically target 17 NRs in several different human osteosarcoma and osteoblast cell lines. The retinoic acid receptor beta (RARb) antagonist LE135, peroxisome proliferator activated receptor gamma (PPARg) antagonist T0070907, liver X receptor (LXR) agonist T0901317 and Rev-Erba agonist SR9011 significantly inhibited the proliferation of malignant osteosarcoma cells (U2OS, HOS-MNNG and Saos-2 cells) but did not inhibit the growth of normal osteoblasts. The effects of these NR modulators on osteosarcoma cells occurred in a dose-dependent manner and were not observed in NR-knockout osteosarcoma cells. These NR modulators also significantly inhibited osteosarcoma growth in vivo and enhanced the antitumour effect of doxorubicin (DOX). Transcriptomic and immunoblotting results showed that these NR modulators may inhibit the growth of osteosarcoma cells by regulating the PI3K/AKT/mTOR and ERK/mTOR pathways. DDIT4, which blocks mTOR activation, was identified as one of the common downstream target genes of these NRs. DDIT4 knockout significantly attenuated the inhibitory effects of these NR modulators on osteosarcoma cell growth. Together, our results revealed that modulators of RARb, PPARg, LXRs and Rev-Erba inhibit osteosarcoma growth both in vitro and in vivo through the mTOR signaling pathway, suggesting that treatment with these NR modulators is a novel potential therapeutic strategy.

## Introduction

Osteosarcoma is the most common type of bone cancer in children and adolescents, and it accounts for 2% of all cancer cases in children aged 0 to 14 and 3% of all cancer cases in adolescents aged 15 to 19 [1, 2]. Tumour resection and nonspecific combination chemotherapy using cisplatin, doxorubicin, and methotrexate remain the primary conventional treatments for osteosarcoma [3]. Patients who do not respond well to these drugs have a poor prognosis. Patients with metastatic or recurrent tumours have a 5-year survival rate of approximately 20%, and this survival rate has remained virtually unchanged over the last three decades [4, 5].

Osteosarcomas are among the most disordered cancers in terms of whole-chromosome and gene copy number changes [6]. Only mutations in the tumour suppressors TP53, RB1, ATRX and CDKN2A have been demonstrated to be associated with osteosarcoma [1, 7]. A limited number of genes are considered to be drivers of osteosarcoma tumours, and there is almost no overlap in the driver genes that have been reported in previous studies [8–10]. However, recent advances in the field of osteosarcoma have revealed new therapeutic opportunities that target several key pathways that regulate the clinical features of oncogenesis and aggressiveness [11]. The inhibition of the PI3K/mTOR pathway, by chemical or genetic approaches, has been demonstrated to be a sensitive and effective approach for osteosarcoma treatment [12–14]. MAPK pathways (ERK, p38 and JNK) play essential roles in cell proliferation, migration, apoptosis and angiogenesis in osteosarcoma [15–17]. Osteosarcoma-associated genes that were identified in a genetic analysis of 119 primary tumours and 134 metastatic nodules are also enriched in the PI3K/mTOR and MAPK pathways [18]. Defining genes that specifically regulate these signaling pathways in osteosarcoma could have a major impact on the identification of new therapeutic targets.

Nuclear receptors (NRs) are a family of transcription factors that localize in cell nuclei, sense specific ligands, and orchestrate a variety of physiological and pathological processes. More than one-third of the 48 known NRs are targets of currently marketed therapeutics, and 20 of the top 200 most frequently prescribed drugs target NRs [19, 20]. NRs have been demonstrated to regulate signaling pathways and biological processes underlying tumorigenesis and cancer progression [21–24]. With the tissue-specific distribution of NRs and their associated molecular networks, each NR performs specific functions in the development of various types of cancer [21]. It remains unclear whether NRs could be potential targets for the treatment of osteosarcoma.

Here, we performed a systemic screen using 29 chemicals that specifically target 17 NRs in three osteosarcoma cell lines (U2OS, HOS-MNNG and Saos-2 cell lines) and one osteoblast cell line (hFOB 1.19 cell line). We found that chemical inhibition of RARb and PPARg (LE135 and T0070907) and activation of LXRs and Rev-Erba (T0901317 and SR9011) significantly suppressed osteosarcoma cell proliferation but not osteoblast proliferation. The effects of these chemicals were almost abolished in the corresponding NR-knockout osteosarcoma cell lines, suggesting that their inhibitory effects are specifically mediated through these NRs. However, none of these NR modulators played a role in promoting apoptosis even in the middle stage of continuous treatment. These NR modulators exerted antitumour effects on osteosarcoma growth in vivo either in the presence or absence of DOX. Our data showed that these chemicals may inhibit osteosarcoma cell proliferation by regulating the PI3K/AKT/mTOR and ERK/mTOR pathways. We also identified DDIT4 as a common target through which these NRs regulate mTOR activity.

## Results

### Identification of NR modulators that inhibit the growth of osteosarcoma cells

**Fig. 1:**
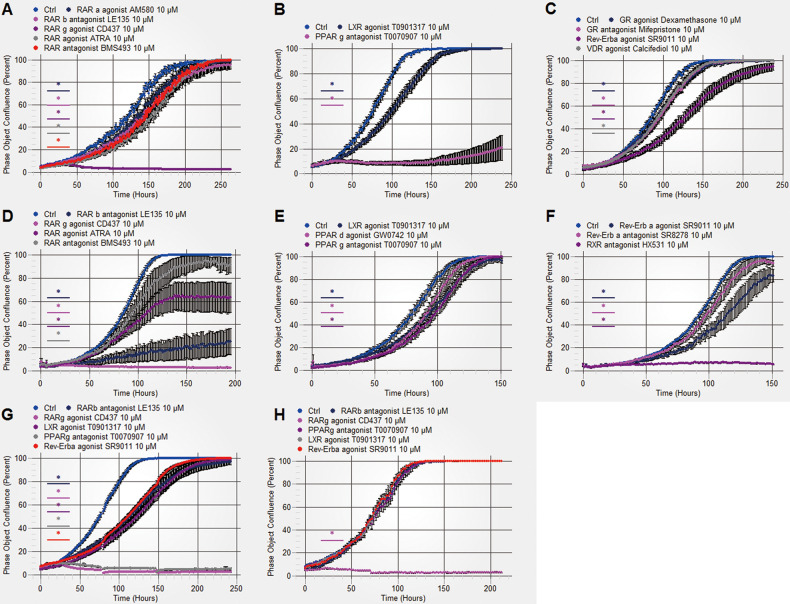
Identification of candidate NR modulators for inhibiting osteosarcoma cell growth. U2OS and HOS-MNNG cells were treated with the 29 indicated NR modulators that target 17 NRs at a concentration of 10 μM. **A**–**C** Eleven NR modulators significantly inhibited U2OS cell growth. **D**–**F** Ten NR modulators significantly inhibited HOS-MNNG cell growth. Saos-2 (**G**) and hFOB 1.19 (**H**) cells were treated with the 5 indicated modulators that targeted a single nuclear receptor at a concentration of 10 μM. The cell confluence was evaluated in real time by the IncuCyte Zoom living cell imaging system. Mean ± SD, *n* = 6; *P < 0.05, the NR modulator group versus the Ctrl group.

The NRs that are commonly expressed in three different osteosarcoma cell lines, namely, the U2OS, HOS-MNNG and Saos-2 cell lines, were identified by analysing our RNA-seq data. Focusing on known and available chemicals that specifically regulate these NRs, we performed a systemic screen using 29 chemicals that target 17 NRs in U2OS and HOS-MNNG cells (Table S1, Fig. 1A–F and Fig. S1). The targeted NRs included RARa, RARb, RARg, PPARa, PPARd, PPARg, LXRa, LXRb, Rev-Erba, THRa, THRb, VDR, AR, ESRRa, GR, MR and RXRa. Eleven chemicals, including the RARa agonist AM580, RARb antagonist LE135, RARg agonist CD437, pan-RAR agonist ATRA, pan-RAR antagonist BMS493, LXR agonist T0901317, PPARg antagonist T0070907, GR agonist dexamethasone, GR antagonist mifepristone, Rev-Erba agonist SR9011 and VDR agonist calcifediol, significantly inhibited U2OS cell growth (Fig. 1A–C). Ten chemicals, including the RARb antagonist LE135, RARg agonist CD437, pan-RAR agonist ATRA, pan-RAR antagonist BMS493, LXR agonist T0901317, PPARd agonist GW0742, PPARg antagonist T0070907, Rev-Erba agonist SR9011, Rev-Erba antagonist SR8278 and RXR antagonist HX531, significantly inhibited HOS-MNNG cell growth (Fig. 1D–F). Among these chemicals, 7 chemicals exerted inhibitory effects on both U2OS and HOS-MNNG cell growth. The exact RAR family members targeted by the pan-RAR modulators ATRA and BMS493 cannot be accurately determined, so we next evaluated the effect of the other 5 chemicals, the RARb antagonist LE135, RARg agonist CD437, LXR agonist T0901317, PPARg antagonist T0070907 and Rev-Erba agonist SR9011, on Saos-2 cell growth. Treatment with these NR modulators also inhibited the growth of Saos-2 cells (Fig. 1G).

To examine the toxic effects of these 5 chemicals on normal osteoblast cells, we treated hFOB 1.19 cells with the same concentration of the 5 chemicals that was used in the osteosarcoma cell assays. The RARg agonist CD437 caused high toxicity in osteoblast cells. None of the other 4 modulators, namely, the RARb antagonist LE135, PPARg antagonist T0070907, LXR agonist T0901317 or Rev-Erba agonist SR9011, affected the growth of normal human cells (Fig. 1H). Our results suggested that 4 NR modulators, namely, the RARb antagonist LE135, PPARg antagonist T0070907, LXR agonist T0901317 and Rev-Erba agonist SR9011, can significantly suppress osteosarcoma cell growth without exerting toxic effects on normal osteoblast cells.

### NR modulators specifically target NRs to affect osteosarcoma cell growth

**Fig. 2:**
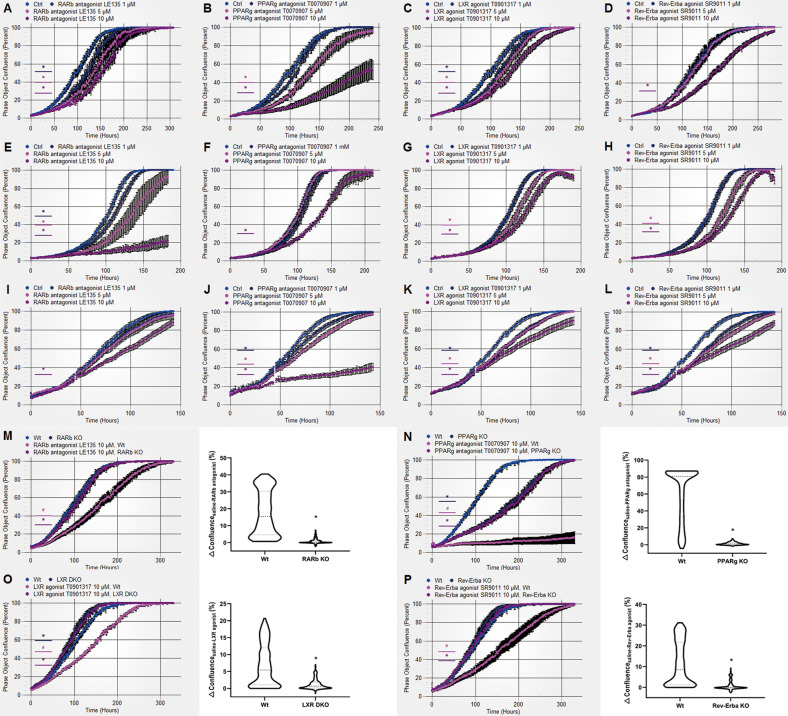
NR modulators inhibited osteosarcoma cell growth by specifically targeting NRs in a dose-dependent manner. **A**–**D** U2OS, **E**–**H** HOS-MNNG, and Saos-2 (**I**–**L**) cells were treated with the RARb antagonist, PPARg antagonist, LXR agonist and Rev-Erba agonist at gradient concentrations (1, 5, or 10 μM). The cell confluence was evaluated in real time by the IncuCyte zoom living cell imaging system. *n* = 6; *P < 0.05, the NR modulator group versus the Ctrl group. **M**–**P** Wild-type (Wt) and NR-knockout (KO) U2OS cells were treated with the corresponding NR modulators at a concentration of 10 μM. The cell confluence was evaluated in real time by the IncuCyte zoom living cell imaging system. Mean ± SD, *n* = 6; *P < 0.05, the NR-KO group versus the Wt group. ^*#*^*P* < 0.05, the NR modulator group versus the saline group. △Confluence represents the difference in confluence between the saline group and the NR modulator group. The violin plots show the distribution of the △Confluence of the indicated genotypes. Paired *t*-tests were used to evaluate the significance. *P < 0.05, the NR KO group versus the Wt group.

We examined whether the 4 chemicals identified above can affect osteosarcoma cells at lower concentrations. Osteosarcoma cells were treated with these chemicals at different concentrations (0.1–10 μM). We found that the NR modulators function in a dose-dependent manner in most osteosarcoma cells (Fig. 2A–L and Fig. S2). Possibly due to the heterogeneity of osteosarcoma cell lines, some osteosarcoma cell lines were only sensitive to high concentrations of certain chemicals; this was true for U2OS cells treated with the Rev-Erba agonist (Fig. 2D), HOS-MNNG cells treated with the PPARg antagonist (Fig. 2F) and Saos-2 cells treated with the RARb antagonist (Fig. 2I).

Then, we investigated the target specificity of the 4 NR modulators in the inhibition of osteosarcoma growth. We deleted RARb, PPARg, LXRs and Rev-Erba in U2OS cells by CRISPR‒Cas9 technology (Fig. S3A–D). The NR-knockout cells were treated with the corresponding modulators, and the growth of these cells was compared with that of wild-type cells under the same treatment conditions (Fig. 2M–P). PPARg knockout dramatically repressed the growth of U2OS cells (Fig. 2N). LXRa and LXRb double knockout slightly accelerated U2OS growth (Fig. 2O). The inhibitory effects of the RARb antagonist LE135, PPARg antagonist T0070907, LXR agonist T0901317 and Rev-Erba agonist SR9011 were all abolished in the corresponding NR-knockout cells (Fig. 2M–P), indicating that the NR modulators specifically target the corresponding NRs to inhibit osteosarcoma cell growth. These results suggest that RARb, PPARg, LXR and Rev-Erba are potential therapeutic targets for osteosarcoma treatment and indicate their roles in regulating osteosarcoma cell growth.

### NR modulators attenuate osteosarcoma cell proliferation

**Fig. 3:**
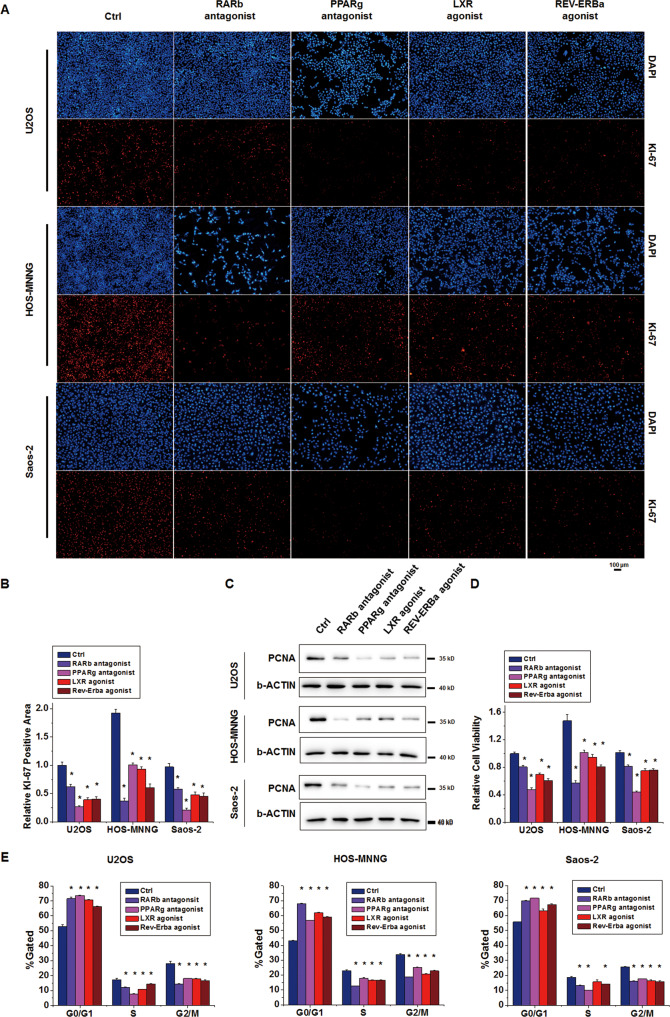
Osteosarcoma cell proliferation was repressed by NR modulator treatments. U2OS, HOS-MNNG and Saos-2 cells were treated with the RARb antagonist, PPARg antagonist, LXR agonist and Rev-Erba agonist at 10 μM for 2 days. **A** The KI-67 protein levels were evaluated by immunofluorescence. Scale bar: 100 μm. **B** The relative KI-67-positive area was normalized to the costained DAPI-positive area. Mean ± SD, *n* = 5; *P < 0.05, the NR modulator group versus the Ctrl group. **C** Cell lysates were collected. The protein levels of PCNA were evaluated by immunoblotting. **D** An MTT viability assay was performed to analyse the cell viability of each group. Mean ± SD, *n* = 5; *P < 0.05, the NR modulator group versus the Ctrl group. **E** Cell cycle analysis was performed after cell collection, fixation and PI staining. The proportions of cells in the G0/G1, S, and M/G2 phases in each group are shown. Mean ± SD, *n* = 3; *P < 0.05, the NR modulator group versus the Ctrl group.

The inhibitory effects of the NR modulators on osteosarcoma cell growth may be attributed to the inhibition of cell proliferation and/or the promotion of apoptosis. To further elucidate the cellular processes that are regulated by these NR modulators, we first evaluated the protein levels of two major proliferation markers, namely, KI-67 and PCNA, in osteosarcoma cells treated with the NR modulators for 2 days. The immunofluorescence and western blotting results showed that the RARb antagonist LE135, PPARg antagonist T0070907, LXR agonist T0901317 and Rev-Erba agonist SR9011 reduced the KI-67 and PCNA protein levels in all three osteosarcoma cell lines (Fig. 3A–C). MTT assays showed that osteosarcoma cell viability also decreased after NR modulator treatment (Fig. 3D). The KI-67 protein is expressed during all active phases of the cell cycle (G1, S, G2, and mitosis), but its expression is almost absent in quiescent cells (G0) [25]. The NR modulators induced cell cycle arrest in the G0/G1 phase in all three osteosarcoma cell lines (Fig. 3E). The proportion of cells in the S phase in most of the osteosarcoma cell lines was decreased by treatment with the 4 chemicals (Fig. 3E). Entrance into the G2/M phase was inhibited by the NR modulators in all the osteosarcoma cell lines (Fig. 3E). We next assessed the effect of the NR modulators on osteosarcoma cell apoptosis. After 5 days of treatment with the NR modulators, the immunofluorescence results showed that only very few cells in each field emitted a positive Cleaved Caspase 3 (CC3) signal in both the control group and the treatment groups (Fig. S4A), and western blotting could not detect CC3 protein expression in any group (Fig. S4B). The level of the apoptosis regulator BAX also did not change after NR modulator treatment (Fig. S4B). Our results showed that NR modulators can cause cell cycle arrest in the quiescent phase and inhibit the proliferation of osteosarcoma cells in the early stage of treatment, but they do not affect apoptosis in osteosarcoma cells even in the middle stage of continuous treatment. These results indicate that the inhibition of cell proliferation rather than the promotion of apoptosis is the key factor that leads to the growth restriction of osteosarcoma cells after treatment with the NR modulators.

### In vivo antitumour effect of the NR modulators

**Fig. 4:**
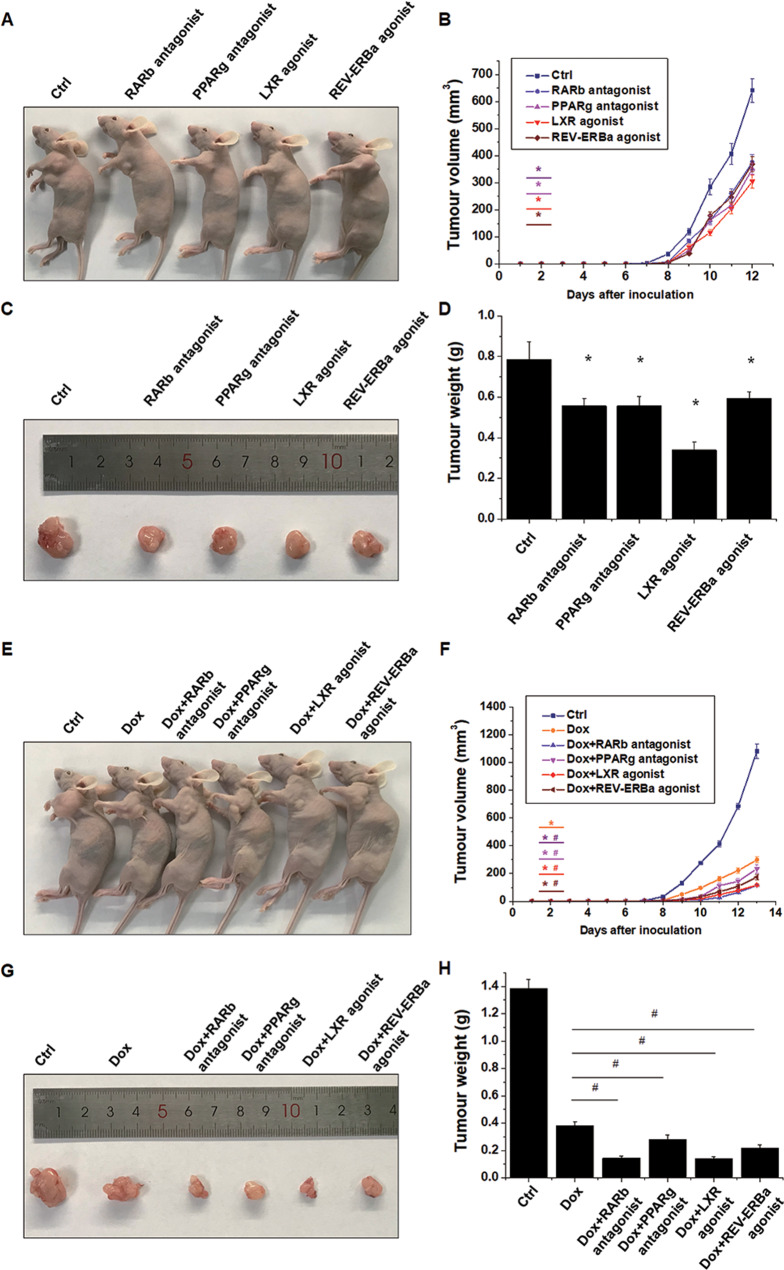
In vivo antitumour effects of the NR modulators. HOS-MNNG cells (5 × 10^6^) were subcutaneously injected into BALB/c nude mice. Beginning on the 3^rd^ day after tumour inoculation, the mice were subcutaneously injected with NR modulators (1 μg dissolved in 100 μl saline) or 100 μl saline in situ every 3 days. **A** Representative images show tumour formation after NR modulator treatment. **B** The tumour volumes were calculated every day after inoculation. **C**, **D** After the mice were sacrificed, the tumours were harvested and weighed. Mean ± SD, *n* = 8; *P < 0.05, the NR modulator group versus the Ctrl group. To evaluate the synergistic effect of NR with DOX, mice were intraperitoneally injected with DOX (5 mg/kg) or saline and subcutaneously injected in situ with the NR modulators (1 μg dissolved in 100 μl saline) or saline every 3 days beginning on the 6^th^ day after tumour inoculation. **E** Representative images show tumour formation in each group. **F** The tumour volumes were calculated every day after inoculation. **G**, **H** After the mice were sacrificed, the tumours were harvested and weighed. Mean ± SD, *n* = 8; *P < 0.05, the DOX or DOX + NR modulator group versus the Ctrl group. ^*#*^*P* < 0.05, the DOX + NR modulator group versus the DOX group.

To test the antitumour effect of the NR modulators in vivo, mice were administered these chemicals in situ every 3 days beginning on the 3rd day after osteosarcoma inoculation. The tumour growth rate was decreased, and the tumour volume and weight were significantly lower in the mice treated with the NR modulators (Fig. 4A–D). We also evaluated the synergistic effects of these chemicals in combination with DOX. The inhibitory effect of DOX on osteosarcoma growth was significantly enhanced by these NR modulators. The tumour volume and weight were significantly lower in all the combination groups compared with DOX alone group (Fig. 4E–H). These data revealed that the NR modulators exert antitumour effects on osteosarcoma growth in vivo.

Compared with the control group, there was no aberrant change in body weight in any of the drug treatment groups (Fig. S5A, E). The activity of aminotransferases (ALT and AST) and the serum concentration of blood urea nitrogen (BUN) and creatinine (CREA) were measured to assess the degree of liver and kidney injury, respectively. Blood biochemical and histological analysis showed that treatment with the NR modulators alone at the concentration used in our study did not cause liver and kidney damage (Fig. S5B–D). DOX treatment resulted in increased serum aminotransferase activity and significant hepatocyte necrosis (Fig. S5F, H). The combination treatment of NR modulators and DOX did not cause kidney injury or the aggravation of DOX-induced liver injury (Fig. S5F–H). Blood biochemical and histological analyses showed that the RARb antagonist and PPARg antagonist significantly ameliorated the liver damage caused by DOX (Fig. S5F, H). These results indicated that treatment with the NR modulators at the concentration we used did not cause significant toxicity in vivo, and some modulators even reduced the liver damage caused by DOX.

### NR modulators suppress osteosarcoma cell proliferation through the mTOR pathway

**Fig. 5:**
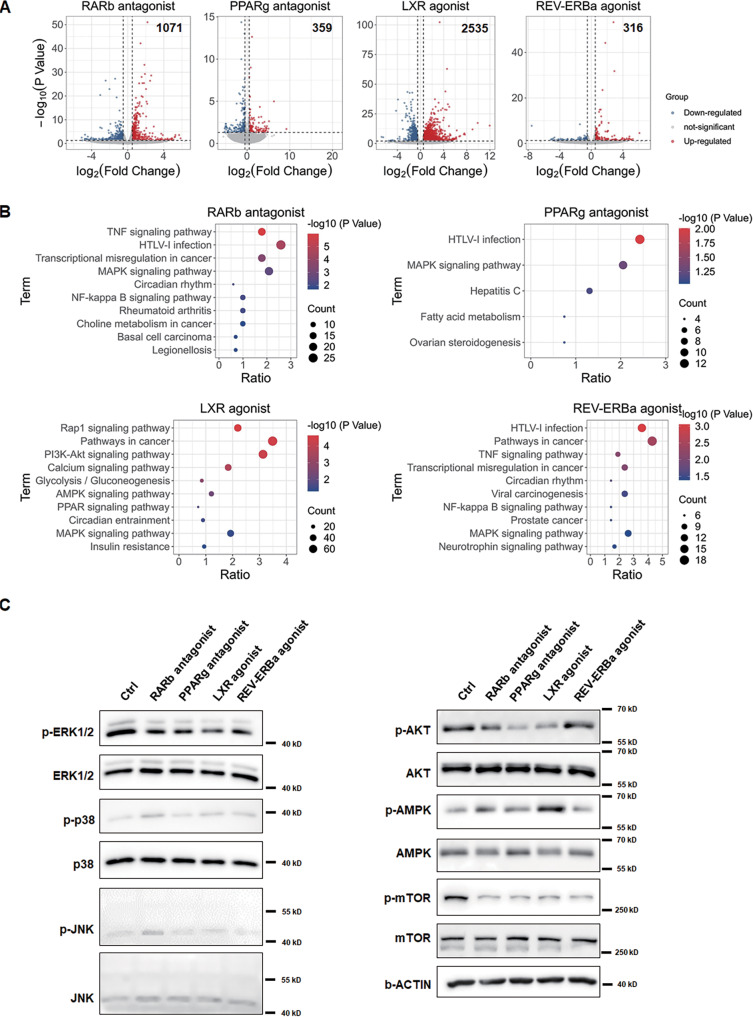
mTOR acted as a core downstream pathway by which the NR modulators inhibited the growth of osteosarcoma. U2OS cells were treated with the RARb antagonist, PPARg antagonist, LXR agonist and Rev-Erba agonist at 10 μM for 24 h. The total RNA of cells was extracted for RNA-seq analysis, and the DEGs of each group were identified (*n* = 3). **A** Volcano plots show all the genes that were statistically upregulated or downregulated and the number of DEGs in each group. **B** Functional enrichment analysis was performed to identify significantly enriched KEGG pathways in each group. **C** Immunoblots show the levels of phosphorylated ERK1/2, p38, JNK, AKT, AMPK and mTOR in cells.

To reveal the key cellular molecules and signaling pathways that participate in the early response to NR modulators, we analysed the transcriptional profile of the osteosarcoma cells after 24 h of treatment. Comparing the RARb antagonist, PPARg antagonist, LXR agonist and Rev-Erba agonist treatment groups with the control group, we identified 1071, 359, 2535 and 316 differentially expressed genes (DEGs), respectively (Fig. 5A). KEGG analysis showed that the pathways regulated by the chemicals were mainly upstream of the mTOR pathway, including the MAPK, TNF, PI3K-AKT, NF-kappa B and AMPK pathways (Fig. 5B). Most of these pathways, as well as the mTOR pathway, have been shown to play key roles in the development of osteosarcoma [12–18]. The western blotting results further confirmed the changes in these signaling pathways after NR modulator treatment (Fig. 5C). The level of phosphorylated ERK1/2 was significantly reduced after treatment with all the chemicals. The signals of the other two MAPK proteins, phosphorylated p38 and JNK, were relatively weak in osteosarcoma cells, despite a subtle increase after RARb antagonist treatment. The PI3K-AKT pathway was also suppressed in cells treated with the RARb antagonist, PPARg antagonist and LXR agonist. The AMPK signaling pathway, which counteracts mTOR activation, was activated by the RARb antagonist, PPARg antagonist and LXR agonist. Suppression of ERK1/2 and AKT and activation of AMPK suggest that the NR modulators exerted an inhibitory effect on the mTOR pathway. The immunoblotting results showed that all the NR modulators dramatically reduced the levels of phosphorylated mTOR in the osteosarcoma cell lines (Fig. 5C). These results suggest that the NR modulators may inhibit the growth of osteosarcoma cells by regulating the PI3K/AKT/mTOR and ERK/mTOR pathways.

### DDIT4 is a key target of NR modulators in the regulation of osteosarcoma proliferation

**Fig. 6:**
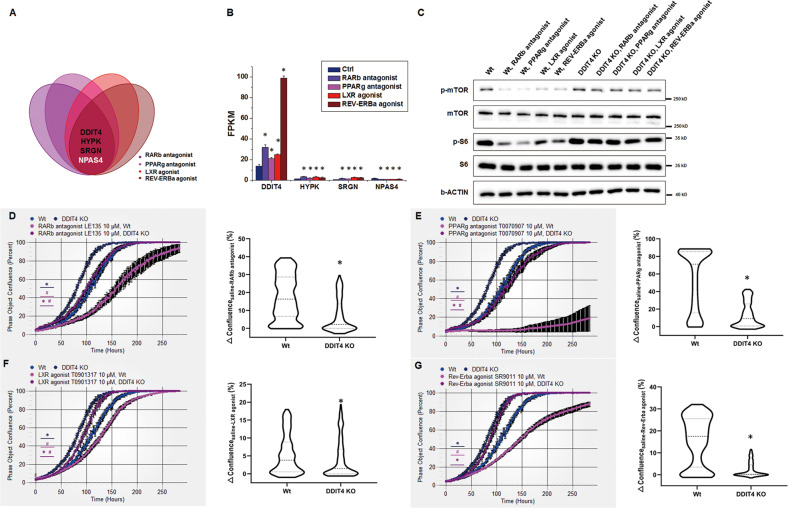
Identification of DDIT4 as a key common target by which the NR modulators inhibited osteosarcoma growth. **A** Plot shows the four genes that were commonly regulated by the NR modulators (upregulated in black, downregulated in white). **B** FPKM of the common targeted genes in each group. Mean ± SD, *n* = 3; *P < 0.05, the NR modulator group versus the Ctrl group. Wt and DDIT4-KO U2OS cells were treated with the RARb antagonist, PPARg antagonist, LXR agonist and Rev-Erba agonist at 10 μM. **C** The levels of phosphorylated mTOR and S6 were evaluated. **D**–**G** Cell growth was evaluated by the IncuCyte zoom living cell imaging system. Mean ± SD, *n* = 6; *P < 0.05, the DDIT4 KO group versus the Wt group. ^*#*^*P* < 0.05, the NR modulator group versus the saline group. △Confluence represents the difference in confluence between the saline group and the NR modulator group. The violin plots show the distribution of the △Confluence of the indicated genotypes. Paired *t*-tests were used to evaluate the significance. *P < 0.05, the DDIT4 KO group versus the Wt group.

DDIT4, HYPK, SRGN and NPAS4 were identified as common downstream targets that are regulated by the NR modulators (Fig. 6A). DDIT4, HYPK and SRGN were upregulated while NPAS4 was downregulated by the treatments (Fig. 6B). Among the four genes, DDIT4 had the highest expression level in osteosarcoma cells, and its expression exhibited the greatest fold change in response to the chemicals. DDIT4 has been demonstrated to regulate cell proliferation and survival by inhibiting the activity of the mTOR complex [26], suggesting its critical role in the inhibition of osteosarcoma by the NR modulators. We generated DDIT4-knockout U2OS cells with CRISPR‒Cas9 technology (Fig. S3E) and treated the cells with the NR modulators. S6 is the downstream effector of mTOR that is related to cell proliferation, and the activation of mTOR leads to an increase in the level of phosphorylated S6 [26]. Here, we found that the NR modulators significantly reduced the level of phosphorylated S6 (Fig. 6C), which was consistent with the inhibitory effect of the NR modulators on osteosarcoma cell proliferation. DDIT4 knockout reversed the NR modulator-induced inhibition of mTOR phosphorylation and further increased the phosphorylation of the downstream protein S6 (Fig. 6C). DDIT4 knockout enhanced osteosarcoma cell growth. The inhibition of wild-type cell growth by the NR modulators was attenuated by DDIT4 knockout (Fig. 6D–G). In particular, the inhibitory effect of the Rev-Erba agonist was almost abolished in DDIT4-knockout cells (Fig. 6G), indicating that the greatest fold changes in DDIT4 expression caused by the Rev-Erba agonist may be the factor that most strongly contributes to its inhibitory effect on osteosarcoma growth. These results revealed DDIT4 as a crucial common target of the NR modulators in the regulation of osteosarcoma cell growth.

## Discussion

Osteosarcoma is one of the most common and challenging malignancies in children and adolescents [1, 2]. In recent years, immunotherapy has provided an effective new approach and breakthrough for cancer therapy; however, its low efficiency in solid tumours, including osteosarcoma, reveals the urgency of a deeper understanding of immunity and the further development of osteosarcoma therapies. Furthermore, traditional chemotherapy is still the first choice for the pharmacological treatment of osteosarcoma [1]. Therefore, the development of therapeutic targets and drugs for osteosarcoma is still one of the most important objectives of osteosarcoma research. In this study, we performed a screen using 29 chemicals that target 17 NRs that are commonly expressed in different types of osteosarcoma cell lines. Four chemicals, namely, the RARb antagonist LE135, PPARg antagonist T0070907, LXR agonist T0901317 and Rev-Erba agonist SR9011, can inhibit the growth of all three osteosarcoma cell lines without affecting the growth of normal osteoblasts. We further demonstrated that the inhibitory effects of these chemicals on osteosarcoma is achieved by specifically targeting corresponding NRs, suggesting that the NRs RARb, PPARg, LXR and Rev-Erba are potential therapeutic targets for osteosarcoma treatment and revealing their roles in regulating osteosarcoma cell growth. These NR modulators also inhibit the formation of osteosarcoma in vivo and enhance the antitumour effect of DOX, suggesting that they may be used as therapeutic agents for osteosarcoma or combined chemotherapy. Whether they can directly enhance the sensitivity of osteosarcoma to DOX remains to be further studied.

A previous study revealed that ATRA can inhibit U2OS cell growth by activating RARa [27]. Our results also showed that both ATRA and the RARa agonist AM580 inhibit U2OS cell growth. However, activation of RARa alone with AM580 does not appear to inhibit the growth of HOS-MNNG cells. In addition to RARa, RARb and RARg are also thought to be activated by ATRA in osteosarcoma cells. We found that the RARb agonist CD2314 does not inhibit the growth of U2OS and HOS-MNNG cells. The RARg agonist CD437 strongly induces the arrests of cell growth in all three osteosarcoma cell lines as well as in osteoblasts. Therefore, in addition to RARa activation, whether ATRA-mediated inhibition of osteosarcoma cell growth may also be partially attributed to RARg activation requires further study. BMS493, which blocks all RAR family members, can inhibit cell growth in osteosarcoma cells. The antagonists of RARa and RARg exhibit no inhibitory effect on osteosarcoma cell growth. We demonstrated that specific blockade of RARb can significantly reduce the growth of all the osteosarcoma cell lines, indicating that RARb may be one of the primary targets by which BMS493 inhibits the growth of osteosarcoma cells. The role of PPARg in cancers remains controversial and may depend on the cancer type and stage [28, 29]. Activating PPARg suppresses the development of most cancers. However, increasing evidence has indicated that PPARg and its activator PGC-1a can also act as tumour promoters [29]. PPARg knockdown or treatment with PPARg antagonists has been shown to suppress hypermalignant subpopulations of breast cancer [30, 31], liver cancer [32, 33], prostate cancer [34] and brain cancer [35–37]. Here, we showed that PPARg antagonist inhibits the development of osteosarcoma both in vitro and in vivo. A previous study showed that LXRa activation inhibits the proliferation of U2OS and Saos-2 cells [38], which is consistent with our results. In this study, we demonstrated that an LXR agonist also significantly represses osteosarcoma tumour growth in vivo, further suggesting that the LXR agonist is a potential agent that can be used for osteosarcoma treatment. Rev-Erba is an essential circadian clock component that acts as a regulator of processes involved in tumorigenesis, including metabolism, proliferation and inflammation [39–42]. Rev-Erba agonists exhibit extensive antitumour activity against a variety of cancer cell lines, including brain, breast, melanoma, leukaemia and colon cancers [43–45], but their effect on osteosarcoma is still unclear. Our results revealed the potency of the Rev-Erba agonist SR9011 as a therapeutic agent for osteosarcoma.

Due to the extensive inhibitory effect of ATRA on the growth of various cancer cells, LE135 is mainly used in cancer research to verify the known inhibitory effect of ATRA on the activation of RARb in cancer cells. For example, LE135 cotreatment can block the RARb activation, growth inhibition, metabolic and metastasis changes caused by ATRA in breast cancer cells [46], melanoma cells [47] and pancreatic cancer cells [48]. However, the effects of LE135 treatment alone on tumour cell growth have not been reported. Here, we demonstrated a significant inhibitory effect of LE135 on osteosarcoma growth both in vitro and in vivo, expanding the potential for LE135 to be used alone or in combination for cancer treatment. In studies suggesting that PPARg has tumour-promoting effects, T0070907 has been proven to inhibit the formation of ERBB2-positive breast cancer cells into tumours [30], the growth of hepatocellular carcinoma cells [32] and the brain metastasis of melanoma [37]. T0901317 exerts antitumour effects in different cancer cells by regulating a variety of intracellular and intercellular processes, including proliferation and metastasis of melanoma [49], lipid metabolism and proliferation of prostate cancer cells [50–52], pyroptosis of colon cancer cells [53], growth of hepatocellular carcinoma cells [54], tumour microenvironment of lung cancer [55], and the proliferation of osteosarcoma, as we demonstrated above. SR9011 can impair stemness and cellular viability by suppressing circadian oscillations in glioma stem cells [45]. SR9011 treatment also inhibits cell growth and promotes apoptosis by regulating autophagy and lipid metabolism in various types of cancer cells [43]. Although these chemicals exert anticancer effects in a variety of cancer cells, the mechanisms are quite different due to the substantial heterogeneity in the gene expression and cellular processes of different types of cancer cells, which may lead to different responses to the same chemicals. There is not enough evidence to claim that these chemicals exert broad spectrum anticancer effects.

mTOR-associated signaling networks have been implicated in the development of various cancer types, including osteosarcoma. Aberrant activation of the mTOR and MAPK pathways is responsible for the proliferative and oncogenic potential of numerous malignant phenotypes of osteosarcoma [56, 57]. The central role of these kinase cascades in the progression of osteosarcoma has been further demonstrated by multiomics studies using a large number of tumour samples. Whole-exome, whole-genome, and RNA-sequencing of 59 pairs of tumour tissues and normal tissues revealed PI3K/AKT/mTOR as the most common pathway that is altered in osteosarcoma [12]. A *Sleeping Beauty* forward genetic screen using 119 primary tumours and 134 metastatic nodules also revealed enrichment in the PI3K/AKT/mTOR and MAPK pathways [18]. Our transcriptomic and immunoblotting data indicated that mTOR- and MAPK-associated pathways, including the ERK1/2, AKT, AMPK and mTOR pathways, respond to the NR modulators in the early stage of treatment and are associated with the inhibition of osteosarcoma growth. DDIT4 inhibits mTOR activation by interacting with the TSC1/2 complex [26]. Enhancing DDIT4 expression has been shown to be an effective strategy for inhibiting the growth of a variety of tumours by repressing mTOR activity and the phosphorylation of the downstream protein S6 [58–64]. Among the four common target genes of the NR modulators in osteosarcoma cells, DDIT4 has the highest expression level and its expression exhibits greatest fold change after treatment with the modulators. DDIT4 knockout accelerates osteosarcoma cell proliferation, which has also been reported in other types of cancer cells [64]. DDIT4 knockout rescues the inhibitory effects of the NR modulators on mTOR phosphorylation, downstream S6 phosphorylation and osteosarcoma cell growth, indicating that the NR/DDIT4/mTOR axis plays a key role in the antitumour effects of the NR modulators. Whether and how these NR modulators alter the activity of mTOR-associated signaling networks and their regulators remain to be further elucidated.

In conclusion, our study revealed that four NR modulators, namely, the RARb antagonist LE135, PPARg antagonist T0070907, LXR agonist T0901317 and Rev-Erba agonist SR9011, effectively inhibit the growth of osteosarcoma in vitro and in vivo. Their inhibitory effects were mainly achieved by specifically targeting the corresponding NRs and repressing abnormal activation of the PI3K/AKT/mTOR and ERK/mTOR pathways in osteosarcoma cells.

## Materials and methods

### Reagents

AM580 (Tocris, #0760, Germany), ER50891 (Tocris, #3823, Germany), CD2314 (Tocris, #3824, Germany), LE135 (Tocris, #2021, Germany), CD437 (Sigma, #C5865, USA), MM11253 (Tocris, #3822, Germany), ATRA (Sigma, #R2625, USA), BMS493 (Sigma, #B6688, USA), GW7647 (Tocris, #1677, Germany), GW6471 (Tocris, #4618, Germany), GW0742 (Tocris, #2229, Germany), GSK3787 (Tocris, #3961, Germany), Troglitazone (Sigma, #T2573, USA), T0070907 (Sigma, #T8703, USA), T0901317 (Sigma, #T2320, USA), SR9238 (Tocris, #5854, Germany), GC1 (Tocris, #4554, Germany), Calcifediol (Tocris, #4036, Germany), SR9011 (Sigma, #SML2067, USA), SR8278 (Tocris, #4463, Germany), CD3254 (Tocris, #3302, Germany), HX531 (Sigma, #SML2170, USA), Testosterone (Aladdin, #T102169, China), Nilutamide (Sigma, #N8534, USA), XCT790 (Sigma, #X4753, USA), Dexamethasone (Sigma, #D4902, USA), Mifepristone (Sigma, #M8046, USA), Corticosterone (Aladdin, #C104537, China), Eplerenone (Sigma, #E6657, USA), Doxorubicin (Solarbio, #D8740, China), Blasticidin (Beyotime, #ST018, China), Puromycin (Beyotime, # ST551, China).

### Cell culture, IncuCyte zoom live imaging system and cell viability analysis

U2OS cells were initially provided by Dr. Eric Zhang at NIBS (Beijing, China). HOS-MNNG (#TCHu167), Saos-2 (#TCHu114) and hFOB 1.19 (#GNHu14) cells were initially purchased from NCACC (Shanghai, China). All the cell lines were regularly tested for contamination by mycoplasma or other pathogens and authenticated using short tandem repeat (STR) profiling. U2OS cells were maintained in DMEM medium (ThermoFisher, USA) supplemented with 10% (v/v) foetal bovine serum (FBS), L-glutamine and 1% penicillin/streptomycin (P/S). HOS-MNNG cells were maintained in MEM (Gibco, USA) supplemented with 10% (v/v) FBS, L-glutamine, sodium pyruvate and 1% P/S. Saos-2 cells were maintained in McCoy’s 5 A medium (Gibco, USA) supplemented with 15% (v/v) FBS, L-glutamine and 1% P/S. hFOB 1.19 cells were maintained in DMEM/F-12 medium (ThermoFisher, USA) supplemented with 10% (v/v) FBS, L-glutamine, 1% P/S and 0.3 mg/ml G418. U2OS, HOS-MNNG and Saos-2 cells were cultured at 37 °C in 5% CO_2_. hFOB 1.19 cells were cultured at 33.5 °C in 5% CO_2_.

The cells seeded in 96-well plates and incubated with NR modulators at 10 μM or a concentration gradient (0.1, 0.3, 1, 5, or 10 μM) for analyses of dose-dependent responses. PBS was added to the culture medium in the control groups. The cells were cultured in the IncuCyte Zoom living cell imaging system (Essen Bioscience, USA) to evaluate cell confluence in real time [65]. For the cell viability MTT assay, cells were seeded at high density in a 96-well plate (5 × 10^4^ cells/well) and treated with the NR modulators at 10 μM for 2 days. The MTT viability assay was performed by using an MTT analysis kit (Beyotime, China).

### In vitro gene deletion by CRISPR‒Cas9 technology

Plasmids expressing sgRNAs were designed and constructed as previously described [66]. The paired sgRNAs for gene knockout were as follows, RARb: 5’-*GACATTGATGTGGTACTCTACGG*-3’ and 5’-*GGTTTGTACACTCGAGGGGGAGG*-3’; PPARg: 5’-*GTGGGAGTGGTCTTCCATTACGG*-3’ and 5’-*AATGGAATGTCTTCGTAATGTGG*-3’; LXRa: 5’-*GCGACGCATGTAGGTGTCCATGG*-3’ and 5’-*GGCCCCTTTTTCCGCTTTTGTGG*-3’; LXRb: 5’-*GCCGCATGAAAGCGTCCATCTGG*-3’ and 5’-*TACAACGTGCTCAGCTGCGAAGG*-3’; Rev-Erba: 5’-*GGTGGCGTCATCACCTACATTGG*-3’ and 5’-*GGCAAGACCCGGCTCGCTCCTTTGG*-3’; DDIT4: 5’-*CAAGGACGAGGGCGAAGAGG*-3’ and 5’-*CTGGGGGTCGGCGACCCGGG*-3’. For the transient transfection experiments, U2OS cells were transfected with 2 μg Cas9 expression plasmid and 1 μg each of two sgRNA expression plasmids (0.5 μg each) for gene deletion by Lipofectamine 3000 (Invitrogen, USA). After 36 to 48 h, the selection antibiotics blasticidin (2 μg/ml) and puromycin (2 μg/ml) were added to cells to select the cells that expressed Cas9 and sgRNAs, and the cells were cultured in media supplemented with these antibiotics for at least one week until cell clones formed. The clones were isolated and collected to evaluate gene deletion by examining the genomic sequences that extend 300–600 bp bilaterally beyond the region between paired sgRNAs.

### Immunofluorescence and cell cycle analyses

Immunofluorescence was performed as described in a previous study [67]. Osteosarcoma cells were seeded at an initial confluence of 30% and were treated with the NR modulators at 10 μM for 2 days. The expression of the proliferation marker KI-67 in the osteosarcoma cells was measured by immunofluorescence staining. The area of positive fluorescence in 5 randomly selected fields in each group was calculated by using ImageJ 1.51j8 (NIH, USA). The relative KI-67-positive area was normalized to the costained DAPI-positive area. Osteosarcoma cells were seeded at an initial confluence of 15% and were treated with the NR modulators at 10 μM for 5 days. The expression of the apoptotic marker Cleaved-Caspase 3 in the osteosarcoma cells was measured by immunofluorescence staining. The antibodies that were used were anti-KI-67 (1:500, Santa Cruz Biotechnology, #sc-23900, USA) and anti-Cleaved-Caspase 3 (1:500, Cell Signaling Technology, #9661, USA) antibodies.

Osteosarcoma cells were treated with the NR modulators for 2 days and harvested for cell cycle analysis. In brief, the cells were fixed in precooled 70% ethanol for 30 min at 4 °C and stained in the dark for 30 min with a solution containing 50 μg/ml propidium iodide (PI). Cells in the different phases of the cell cycle were then identified by using a FACScan flow cytometer (BD Biosciences, USA).

### Mouse xenograft models

BALB/c nude mice (4–6 weeks old, female) were purchased from GemPharmatech (Nanjing, China) and raised at CAM-SU GRC, Soochow University. All the animal care and procedures were performed in accordance with the guidelines of CAM-SU GRC, Soochow University (CAM-SU-AP#: YX-2019-1). The mice were randomly divided into different groups. To generate murine subcutaneous tumours, HOS-MNNG cells (5 × 10^6^) were subcutaneously injected into the flanks of the female BALB/c nude mice in each group (*n* = 8). Beginning on the 3^rd^ day after tumour inoculation, the mice were subcutaneously injected in situ with the NR modulators (1 μg dissolved in 100 μl saline) or 100 μl saline as a control every 3 days to evaluate the antitumour effects of the NR modulators. To evaluate the synergistic effect of NR with DOX, the mice were intraperitoneally injected with DOX (5 mg/kg) and subcutaneously injected in situ with the NR modulators (1 μg dissolved in 100 μl saline) or an equal volume of saline as a control every 3 days beginning on the 6^th^ day after tumour inoculation. Tumour volumes were calculated with the following formula: length x width^2^ x π/6 [68]. The mice were weighed every day during the experiments (*n* = 5). The tumours were harvested and weighed after the mice were sacrificed (*n* = 8). The sera, livers and kidneys were harvested to evaluate the toxicity of the chemicals (*n* = 5) as previously described [69]. The investigator was blinded to the group allocation of the animals during the experiment. No statistical method was used to predetermine the sample size for the xenograft mouse experiment, and the sample size was based on previous experimental observations. No data were excluded from the analysis.

### RNA-seq and differentially expressed gene analyses

Untreated U2OS, HOS-MNNG and Saos-2 cells were harvested and used to identify coexpressed NR genes in the osteosarcoma cell lines. U2OS cells treated with the NR modulators for 24 h were harvested to evaluate the primary and early effects of the NR modulators on gene expression at the whole transcriptome level. Total RNA was extracted from the cells with TRIzol Reagent (Thermo Fisher, USA). The quality of the total RNA was determined using an Agilent 2200 TapeStation, and RNA-seq was performed on an Illumina NovaSeq 6000 platform with PE 150 bp reads at Genewiz, Suzhou, China. Paired-end clean reads were aligned to the human reference genome hg38 using RSEM1.2.8, and expression values were normalized by FPKM. An FPKM value of 0.1 was set as the threshold for determining whether a gene was expressed. The R statistical package software DEseq2 was used to identify differentially expressed genes. Genes with corrected *p* < 0.05 and |log_2_(Fold Change)| ≥ 0.5 were considered to be significantly differentially expressed genes (DEGs). In addition, functional enrichment analysis with DAVID (https://david.ncifcrf.gov/home.jsp) was performed to identify significantly enriched KEGG terms.

### Western blotting analysis

Osteosarcoma cells were treated with the NR modulators for 2 days, and the protein levels of the proliferation marker PCNA were measured by western blotting. The protein levels of Cleaved-Caspase 3, Caspase 3 and BAX in the cells were measured after 5 d of NR modulator treatment. The level of total and phosphorylated MAPK (ERK, p38 and JNK), AKT, AMPK, mTOR and S6 in cells treated with the NR modulators for 24 h were evaluated. Western blots were performed as previously described [66]. The antibodies that were used were anti-PCNA (1:500, Santa Cruz Biotechnology, #sc-56, USA), anti-Cleaved-Caspase 3 (1:1000, Cell Signaling Technology, #9661, USA), anti-Caspase 3 (1:1000, Cell Signaling Technology, #9662, USA), anti-BAX (1:1000, Cell Signaling Technology, #2772, USA), anti-p-ERK1/2 (1:1000, Cell Signaling Technology, #9101, USA), anti-ERK1/2 (1:1000, Cell Signaling Technology, #9102, USA), anti-p-p38 (1:1000, Cell Signaling Technology, #4511, USA), anti-p38 (1:1000, Cell Signaling Technology, #8690, USA), anti-p-JNK (1:1000, Cell Signaling Technology, #4668, USA), anti-JNK (1:1000, Cell Signaling Technology, #9252, USA), anti-p-AKT (1:1000, Cell Signaling Technology, #4060, USA), anti-AKT (1:1000, Cell Signaling Technology, #4685, USA), anti-p-AMPK (1:1000, Cell Signaling Technology, #2535, USA), anti-AMPK (1:1000, Cell Signaling Technology, #2532, USA), anti-p-mTOR (1:1000, Cell Signaling Technology, #5536, USA), anti-mTOR (1:1000, Cell Signaling Technology, #2983, USA), anti-p-S6 (1:1000, Cell Signaling Technology, #2215, USA), anti-S6 (1:1000, Cell Signaling Technology, #2217, USA) and anti-b-ACTIN (1:2000, Sigma, #A5441, USA).

### Statistical analysis

In all the experiments, the data are reported as the mean ± SD of at least 3 replicates per group. All the statistical analyses were performed using two-sided Student’s *t*-tests, paired *t*-tests or two-way ANOVAs using GraphPad Prism 8 (GraphPad Software Inc., USA). *p* < 0.05 was considered statistically significant. All the data that met the assumptions of the tests and statistical tests were justified as appropriate. All the experiments were repeated at least three times.

## Supplementary information

Original Data File

Reproducibility Checklist

Supplemental Figure 1

Supplemental Figure 2

Supplemental Figure 3

Supplemental Figure 4

Supplemental Figure 5

Supplemental_Table_S1

Supplemental_Table_S2

## Data Availability

RNA-seq data that support the findings of this study have been deposited in the NCBI’s Sequence Read Archive (SRA) with the accession number PRJNA865815. The data supporting the findings of this study are available within the article and its supplementary information files. Source data are provided in this paper.
