# Correction: Nuclear receptor modulators inhibit osteosarcoma cell proliferation and tumour growth by regulating the mTOR signaling pathway

**DOI:** 10.1038/s41419-025-07409-2

**Published:** 2025-02-10

**Authors:** Baoshi Yuan, Kexin Shi, Juanmin Zha, Yujia Cai, Yue Gu, Kai Huang, Wenchang Yue, Qiaocheng Zhai, Ning Ding, Wenyan Ren, Weiqi He, Ying Xu, Tao Wang

**Affiliations:** 1 Cambridge-Su Genomic Resource Center, Suzhou medical college of Soochow University, Suzhou, Jiangsu 215123 China; 2 Ministry of Education Key Laboratory of Biosystems Homeostasis and Protection and Innovation Center for Cell Signaling Network, Life Sciences Institute, Zhejiang University, Hangzhou, Zhejiang 310030 China; 3 Department of Oncology, The First Affiliated Hospital of Soochow University, Suzhou, Jiangsu 215006 China; 4 Department of Urology, The First Affiliated Hospital of Soochow University, Suzhou, Jiangsu 215006 China; 5 Department of Orthopaedics, the Second Affiliated Hospital of Soochow University, Suzhou, Jiangsu 215004 China

**Keywords:** Cancer therapy, Sarcoma

Correction to: *Cell Death & Disease* 10.1038/s41419-022-05545-7, published online 21 January 2023

The acknowledgment “We are very grateful to Dr. Eric Zhang for providing the U2OS cells. This work was supported by the grants from National Natural Science Foundation of China (31601022), National Key R&D Program of China (2018YFA0801104), and The Livelihood and Technology Program of Suzhou City (N324560122).” has been updated to “ “We are very grateful to Dr. Eric Zhang for providing the U2OS cells. This work was supported by the grants from National Natural Science Foundation of China (31601022), National Key R&D Program of China (2018YFA0801104) and The Livelihood and Technology Program of Suzhou City (SKY2022106).”

The original article has been corrected.

