# Supplementary material for: Nuclear receptor modulators inhibit osteosarcoma cell proliferation and tumour growth by regulating the mTOR signaling pathway
Source: Cell Death Dis. 2023 Jan 21;14(1):51. doi: 10.1038/s41419-022-05545-7 (PMC9867777; doi:10.1038/s41419-022-05545-7)
Supplement: Supplementary file 1 — Original Data File [file 41419_2022_5545_MOESM1_ESM.pptx]

## Slide 1
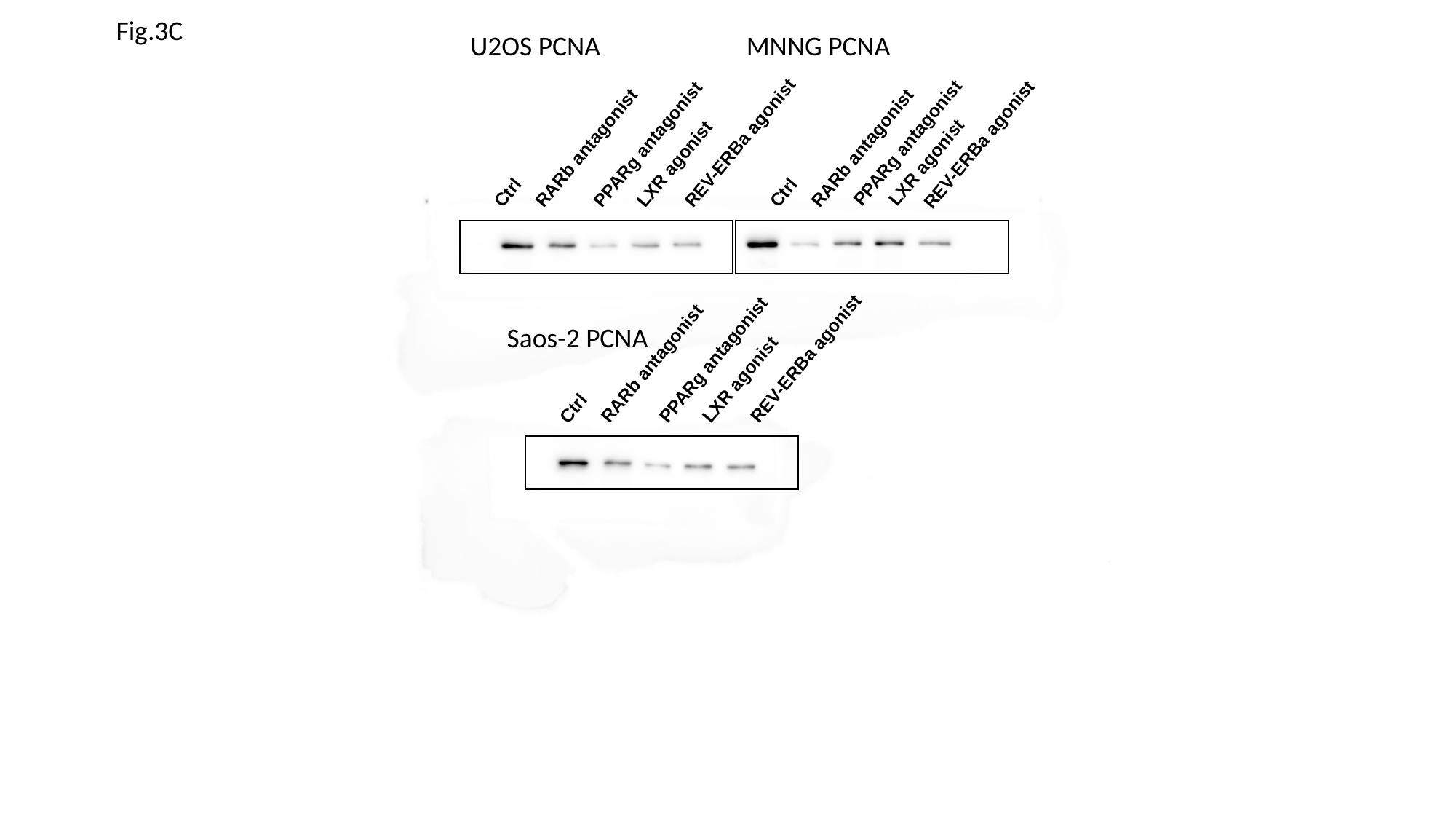

Fig.3C
U2OS PCNA
MNNG PCNA
PPARg antagonist
LXR agonist
RARb antagonist
PPARg antagonist
LXR agonist
REV-ERBa agonist
RARb antagonist
REV-ERBa agonist
Ctrl
Ctrl
Saos-2 PCNA
RARb antagonist
PPARg antagonist
LXR agonist
REV-ERBa agonist
Ctrl

## Slide 2
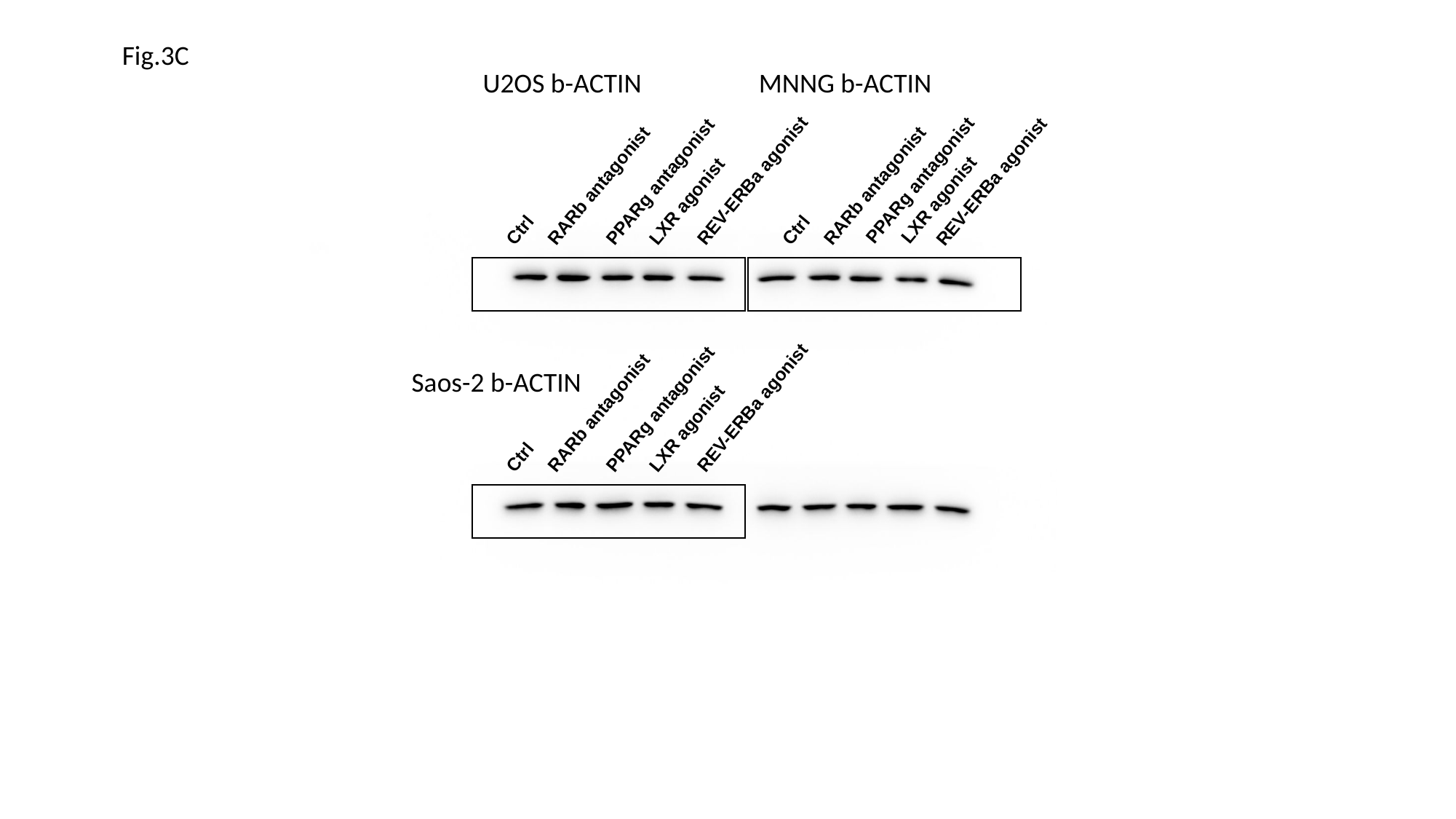

Fig.3C
U2OS b-ACTIN
MNNG b-ACTIN
PPARg antagonist
LXR agonist
RARb antagonist
PPARg antagonist
LXR agonist
REV-ERBa agonist
RARb antagonist
REV-ERBa agonist
Ctrl
Ctrl
Saos-2 b-ACTIN
RARb antagonist
PPARg antagonist
LXR agonist
REV-ERBa agonist
Ctrl

## Slide 3
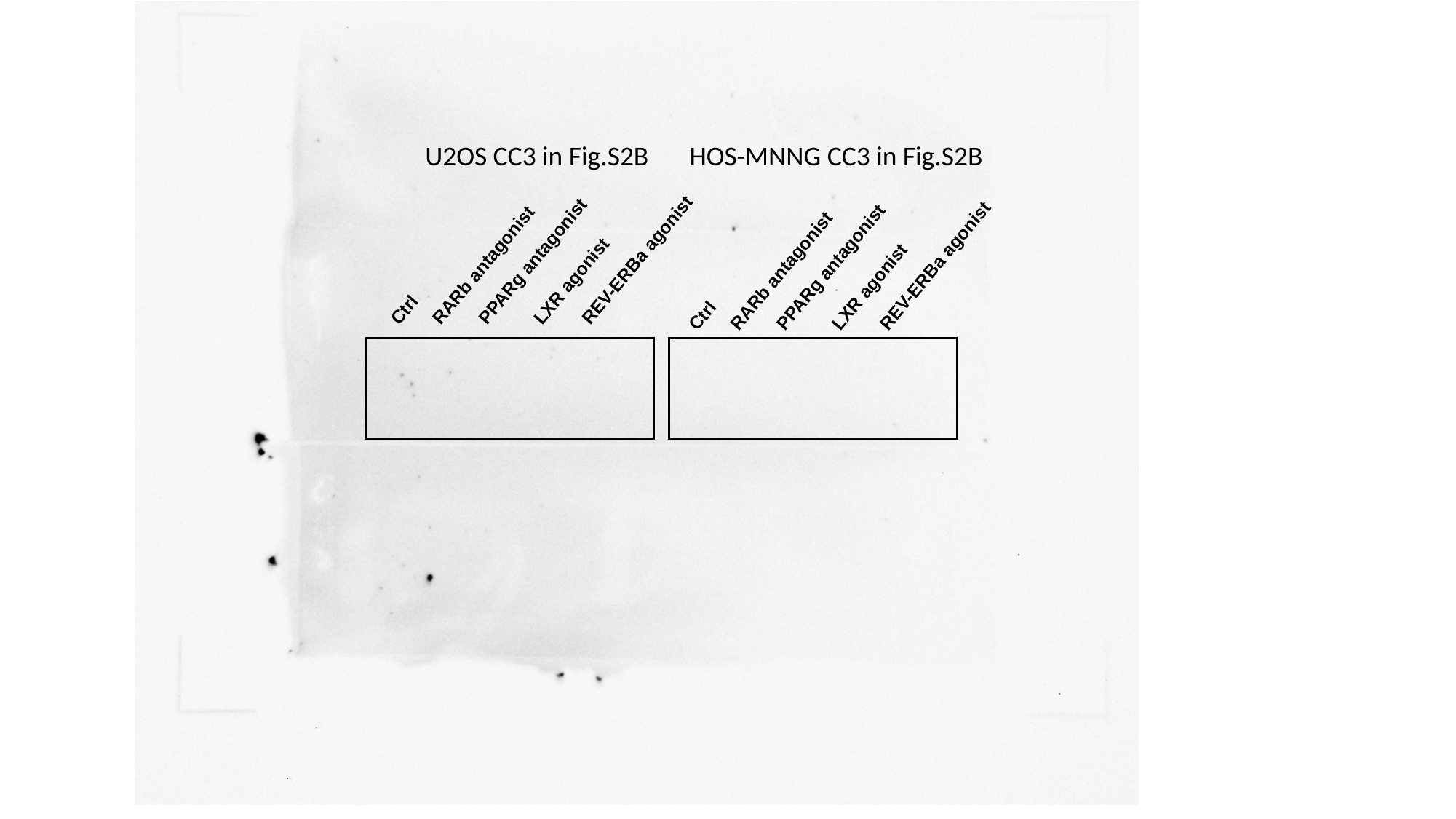

HOS-MNNG CC3 in Fig.S2B
U2OS CC3 in Fig.S2B
RARb antagonist
PPARg antagonist
LXR agonist
REV-ERBa agonist
RARb antagonist
PPARg antagonist
LXR agonist
REV-ERBa agonist
Ctrl
Ctrl

## Slide 4
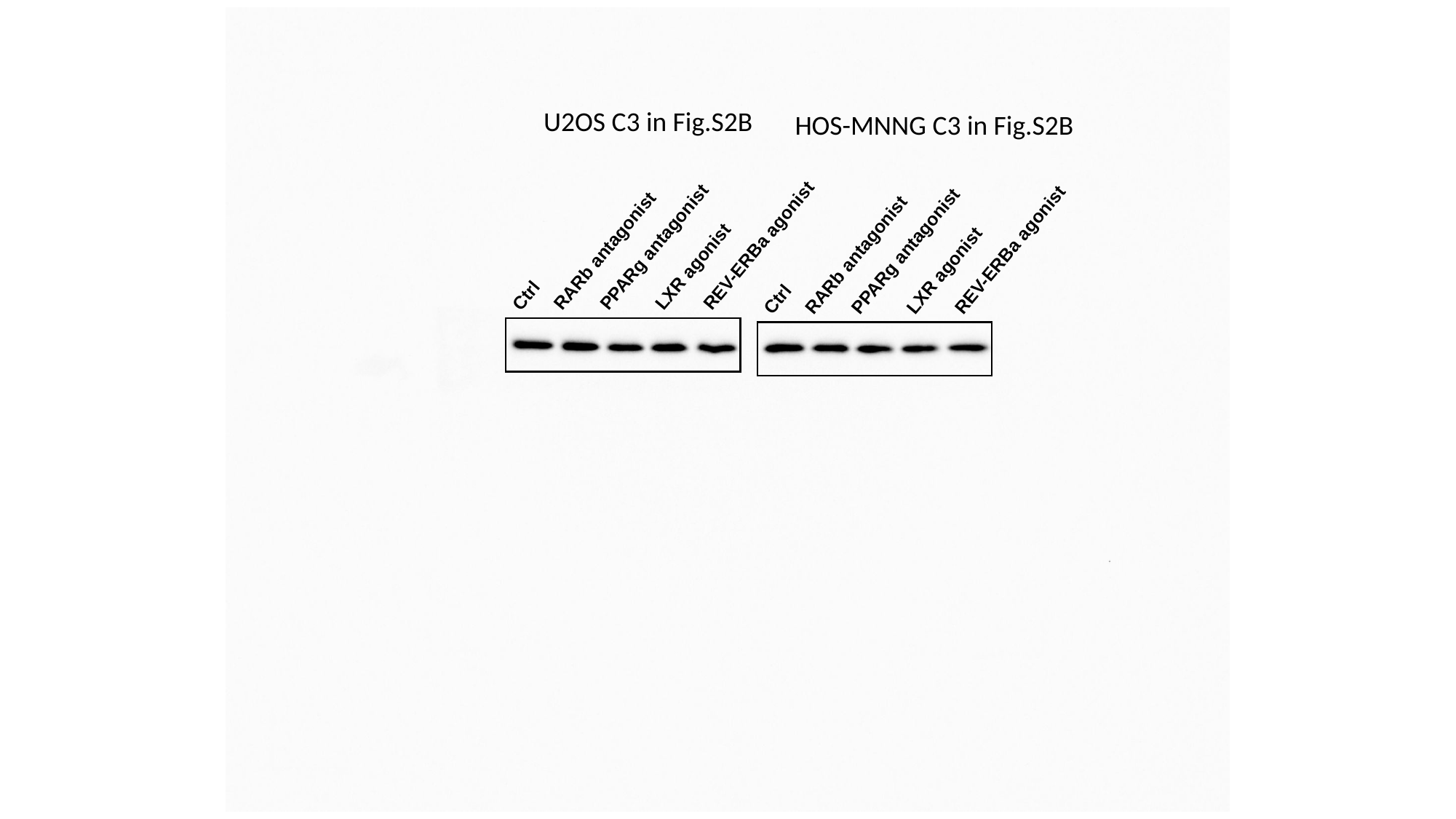

U2OS C3 in Fig.S2B
HOS-MNNG C3 in Fig.S2B
RARb antagonist
PPARg antagonist
LXR agonist
REV-ERBa agonist
RARb antagonist
PPARg antagonist
LXR agonist
REV-ERBa agonist
Ctrl
Ctrl

## Slide 5
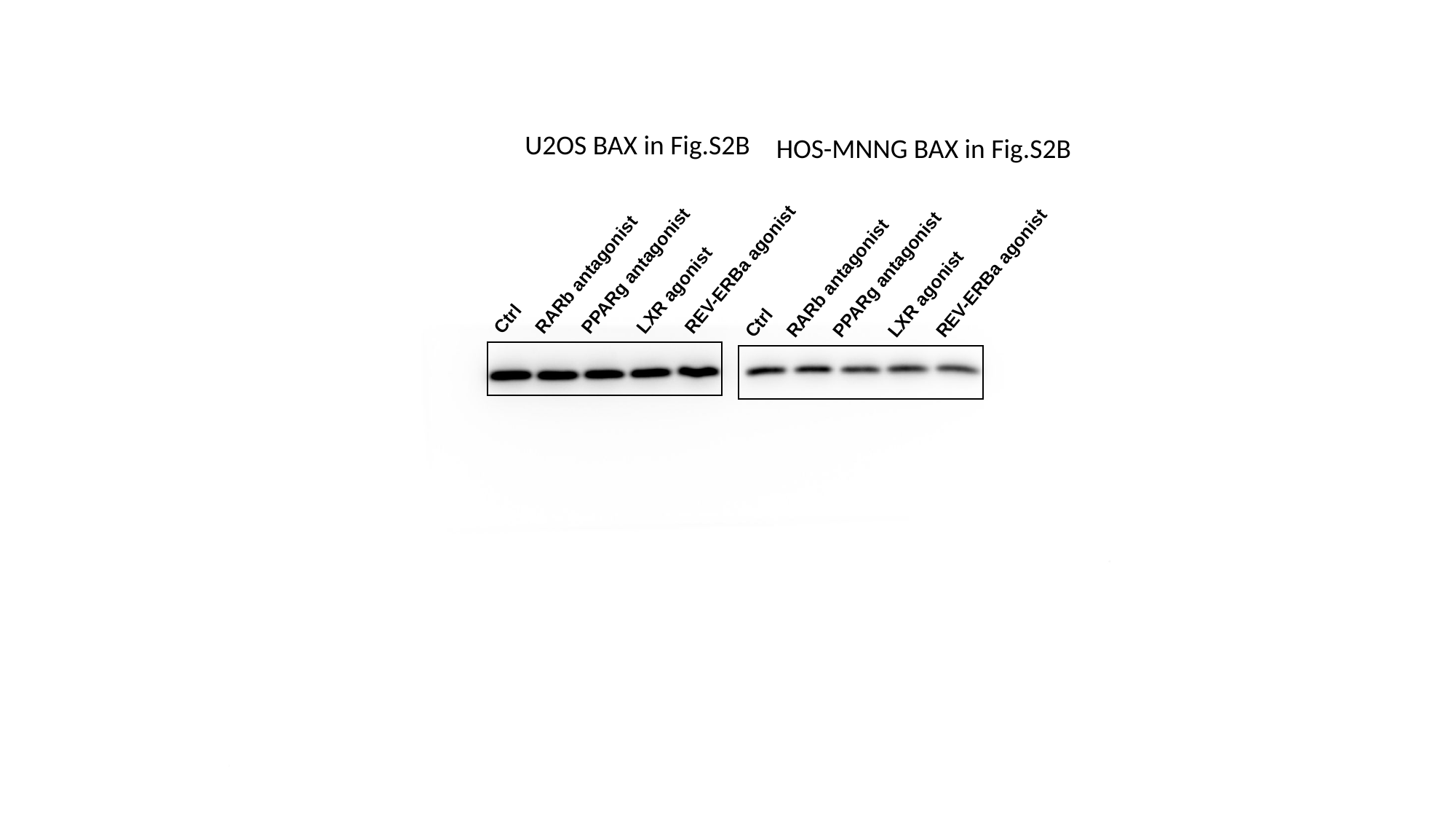

U2OS BAX in Fig.S2B
HOS-MNNG BAX in Fig.S2B
RARb antagonist
PPARg antagonist
LXR agonist
REV-ERBa agonist
RARb antagonist
PPARg antagonist
LXR agonist
REV-ERBa agonist
Ctrl
Ctrl

## Slide 6
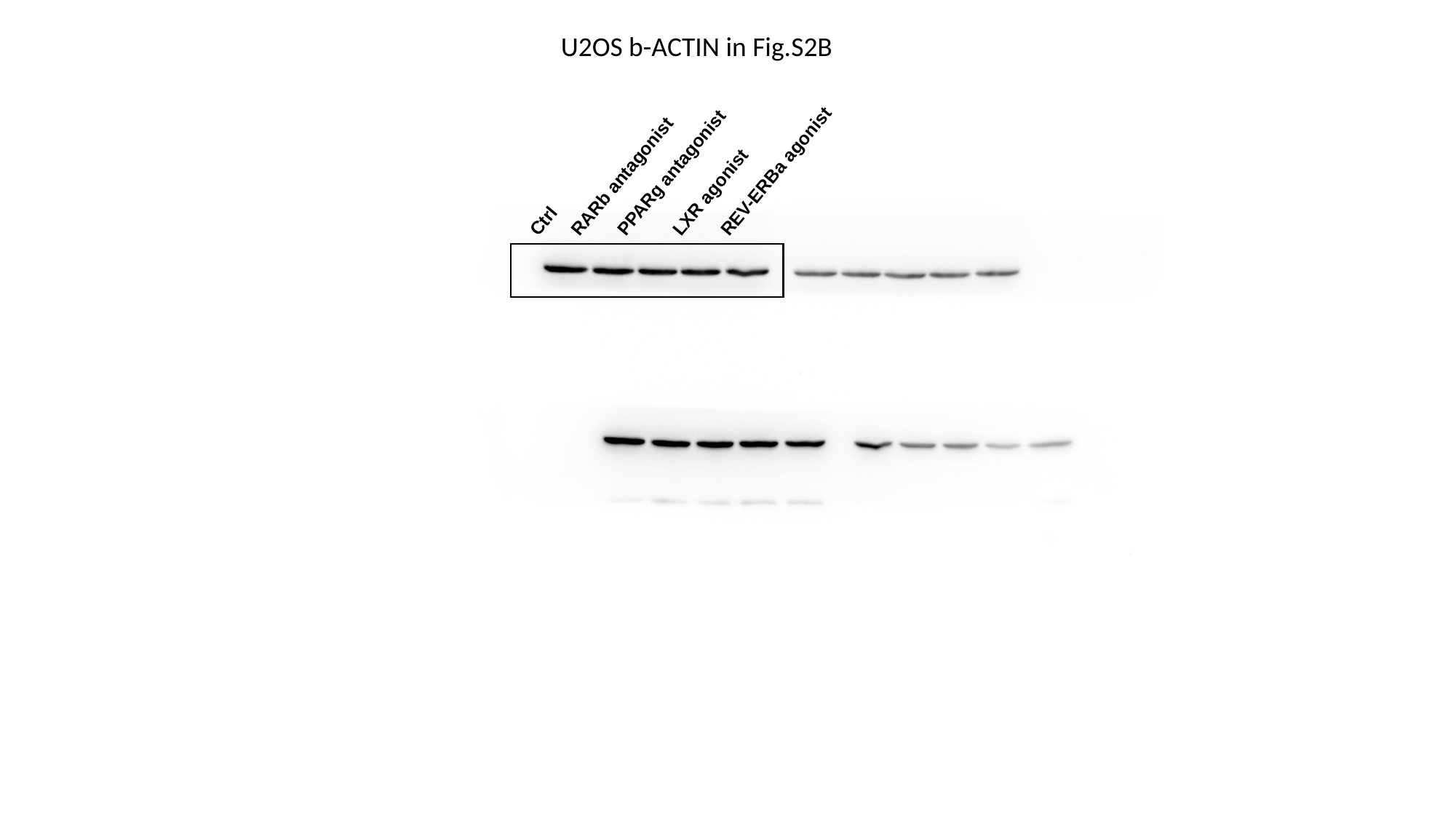

U2OS b-ACTIN in Fig.S2B
RARb antagonist
PPARg antagonist
LXR agonist
REV-ERBa agonist
Ctrl

## Slide 7
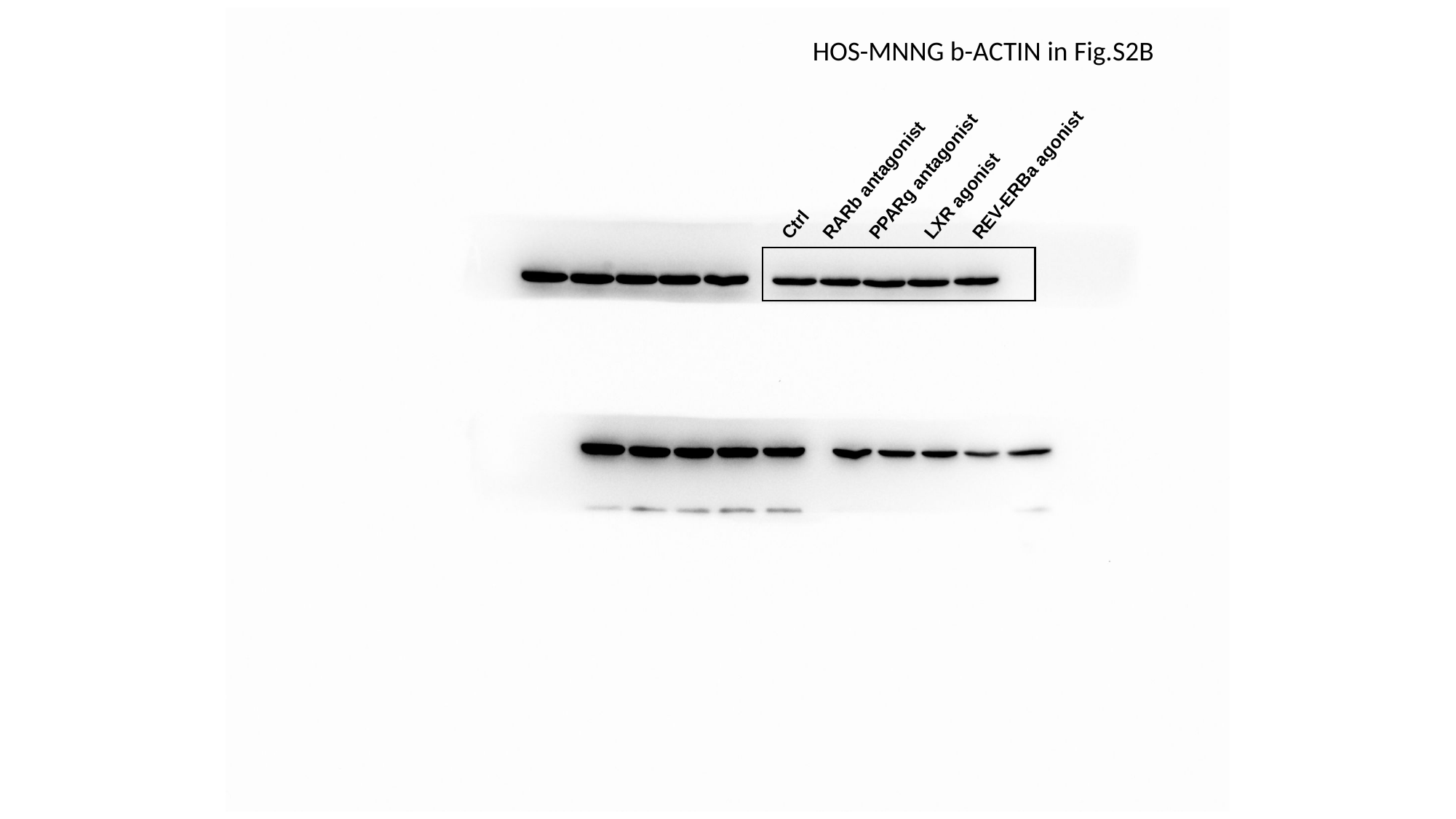

HOS-MNNG b-ACTIN in Fig.S2B
RARb antagonist
PPARg antagonist
LXR agonist
REV-ERBa agonist
Ctrl

## Slide 8
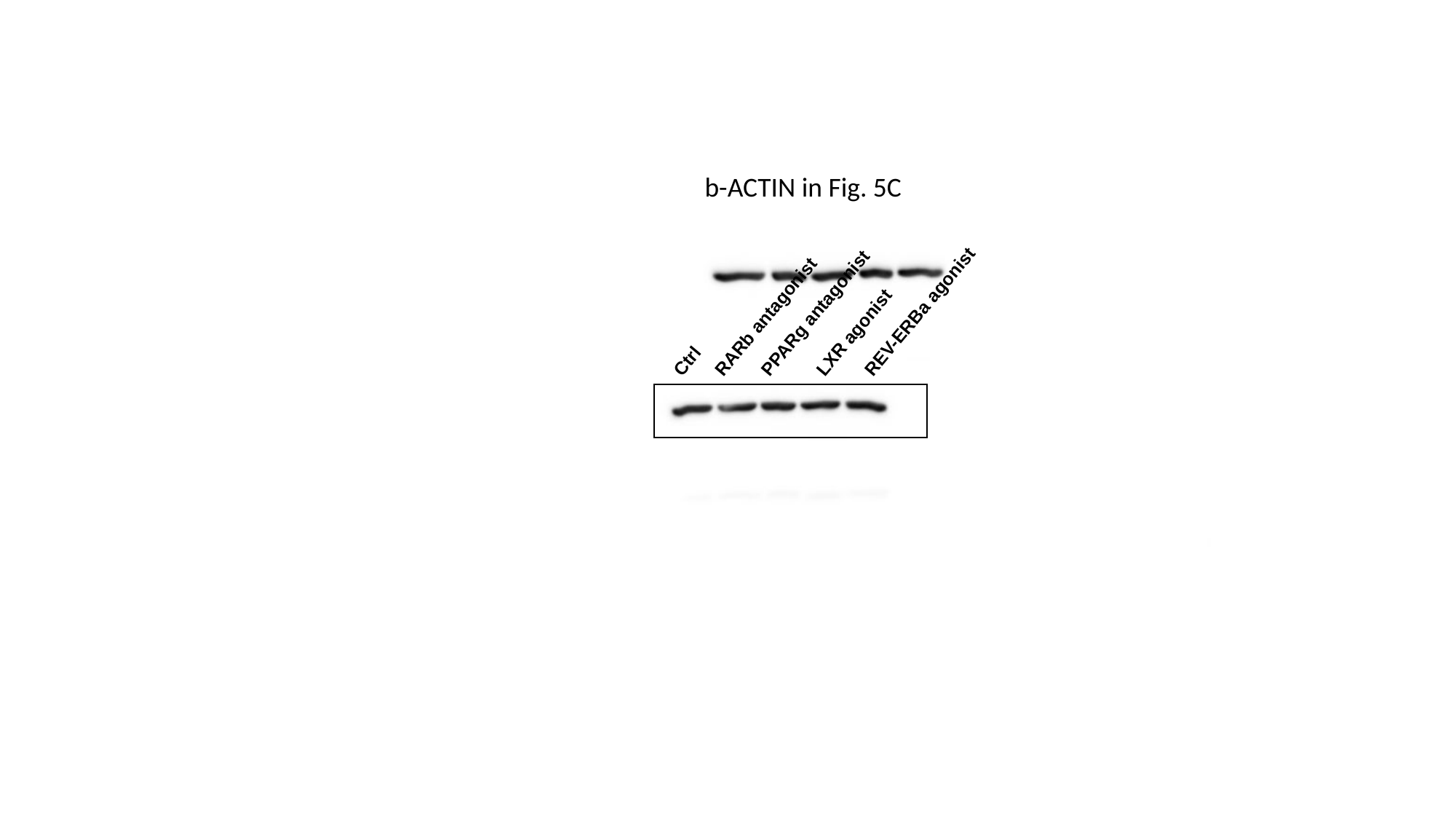

b-ACTIN in Fig. 5C
RARb antagonist
PPARg antagonist
LXR agonist
REV-ERBa agonist
Ctrl

## Slide 9
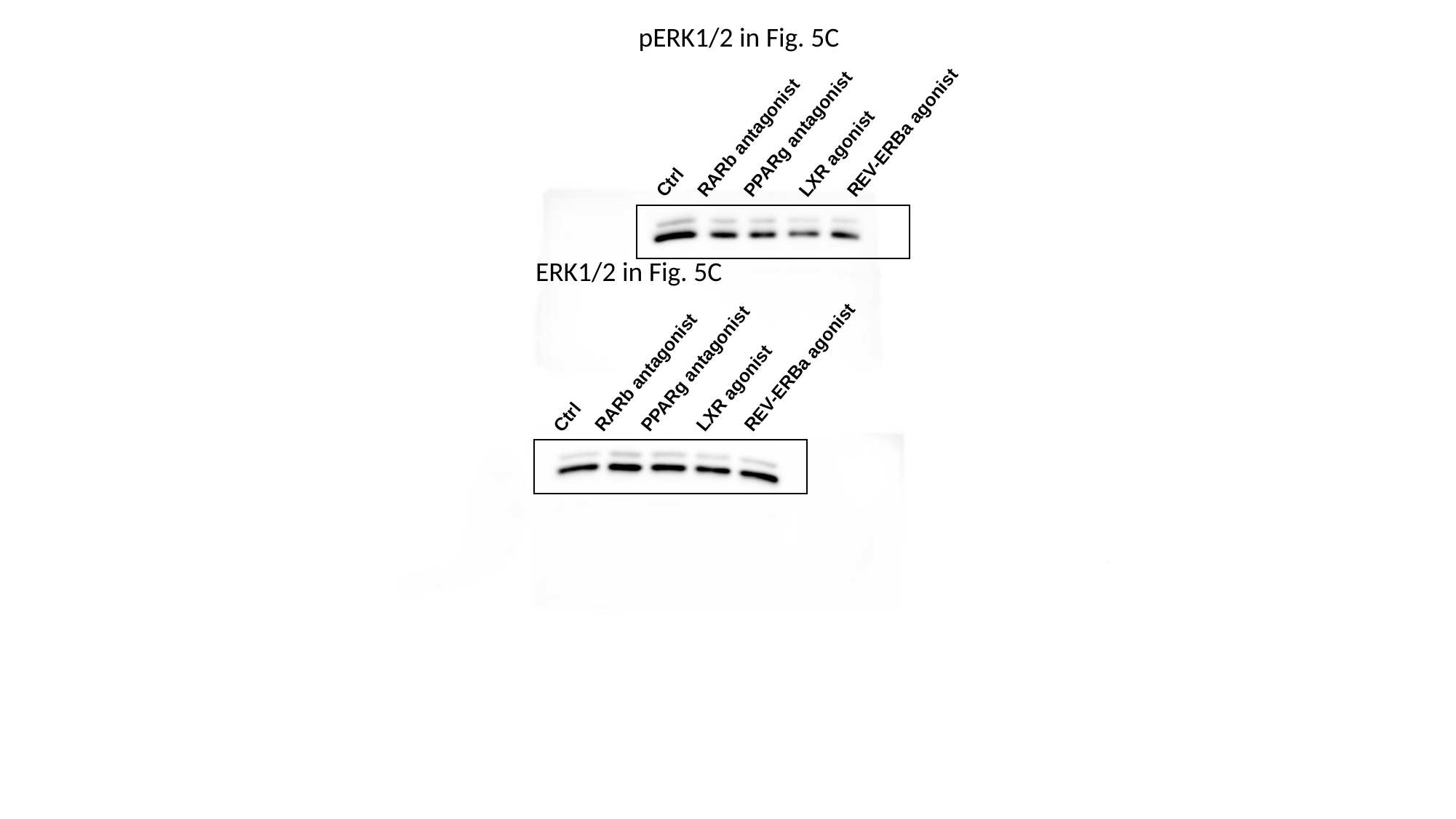

pERK1/2 in Fig. 5C
RARb antagonist
PPARg antagonist
LXR agonist
REV-ERBa agonist
Ctrl
ERK1/2 in Fig. 5C
RARb antagonist
PPARg antagonist
LXR agonist
REV-ERBa agonist
Ctrl

## Slide 10
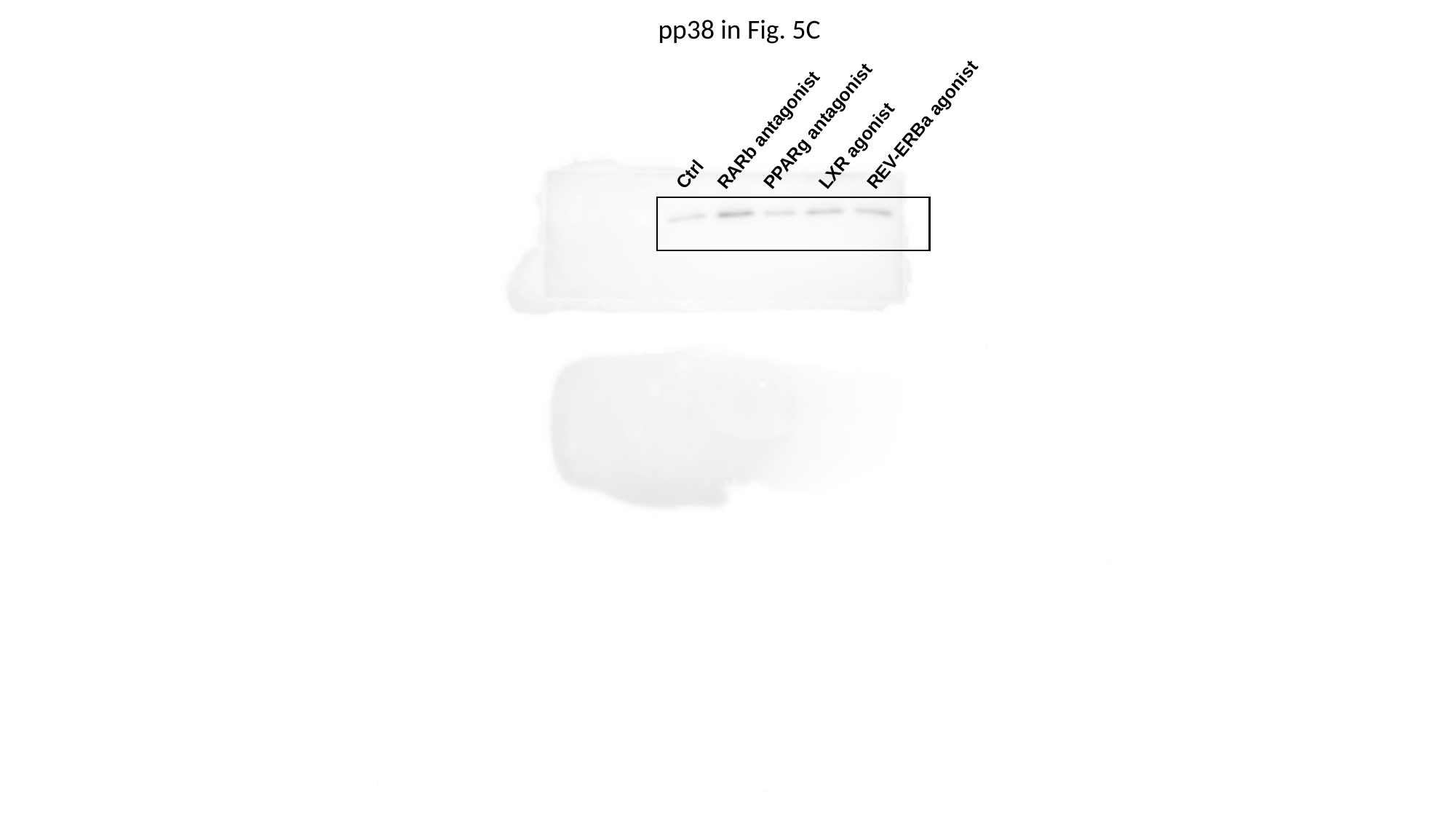

pp38 in Fig. 5C
RARb antagonist
PPARg antagonist
LXR agonist
REV-ERBa agonist
Ctrl

## Slide 11
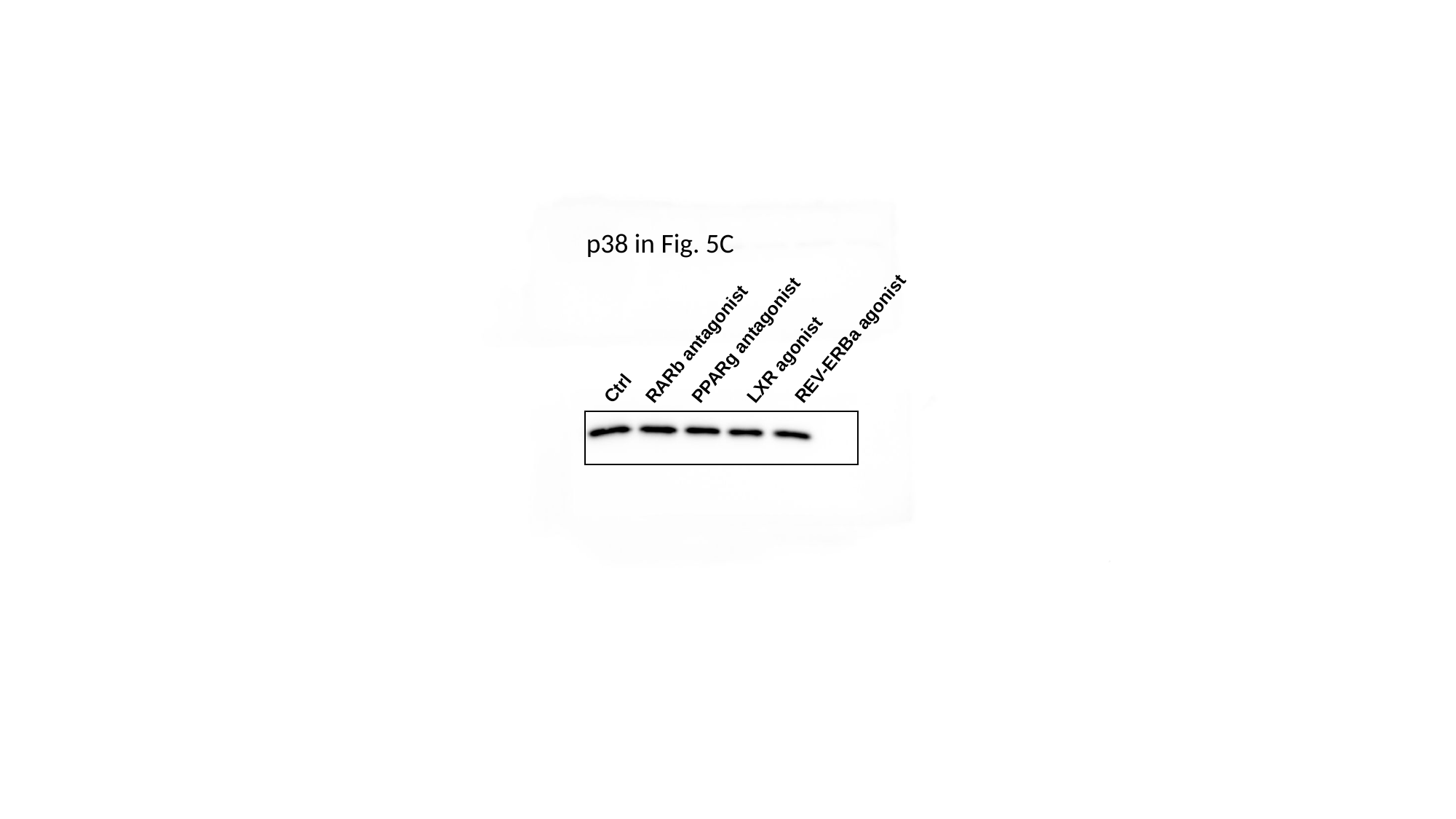

p38 in Fig. 5C
RARb antagonist
PPARg antagonist
LXR agonist
REV-ERBa agonist
Ctrl

## Slide 12
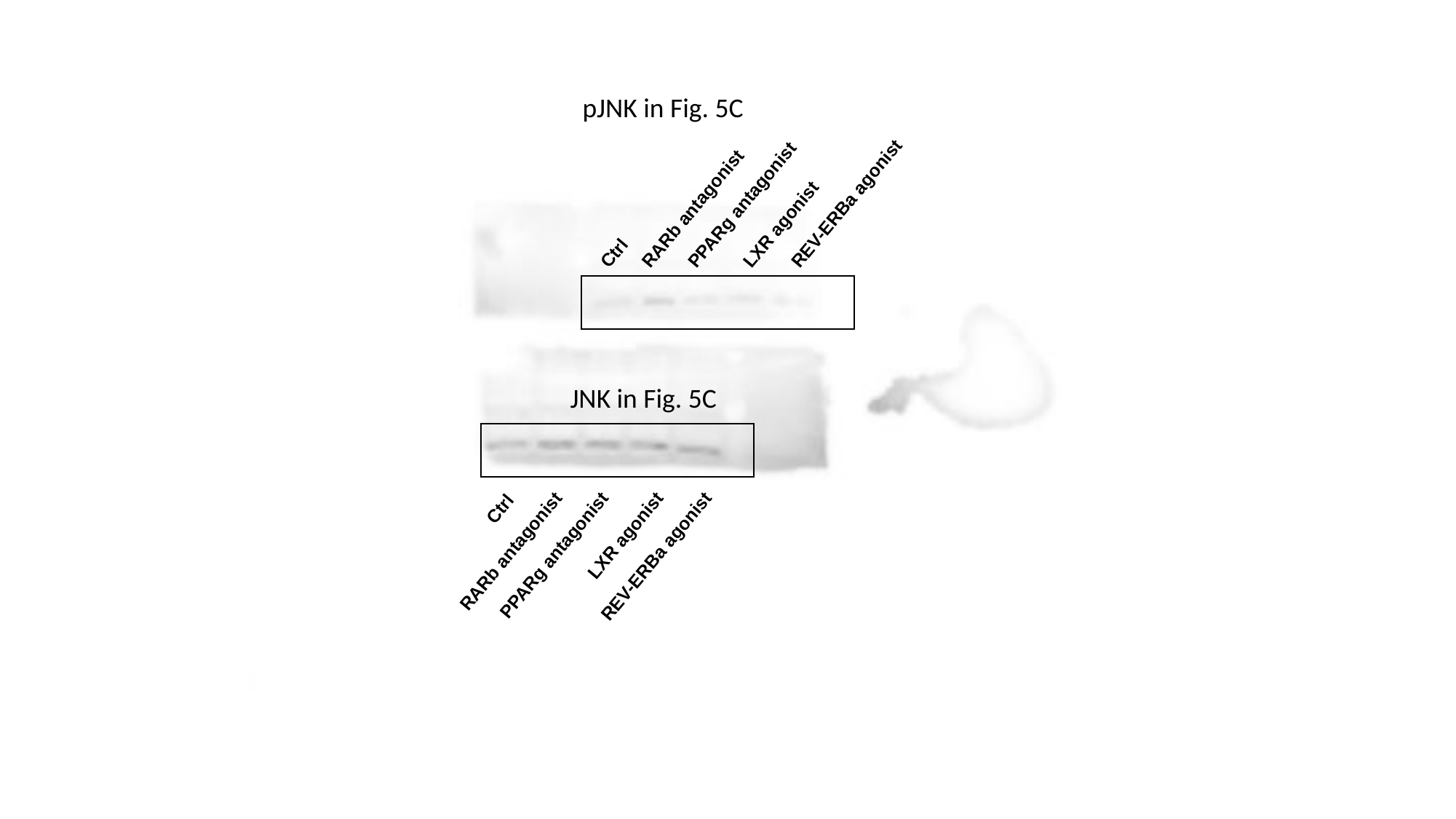

pJNK in Fig. 5C
RARb antagonist
PPARg antagonist
LXR agonist
REV-ERBa agonist
Ctrl
JNK in Fig. 5C
Ctrl
RARb antagonist
PPARg antagonist
LXR agonist
REV-ERBa agonist

## Slide 13
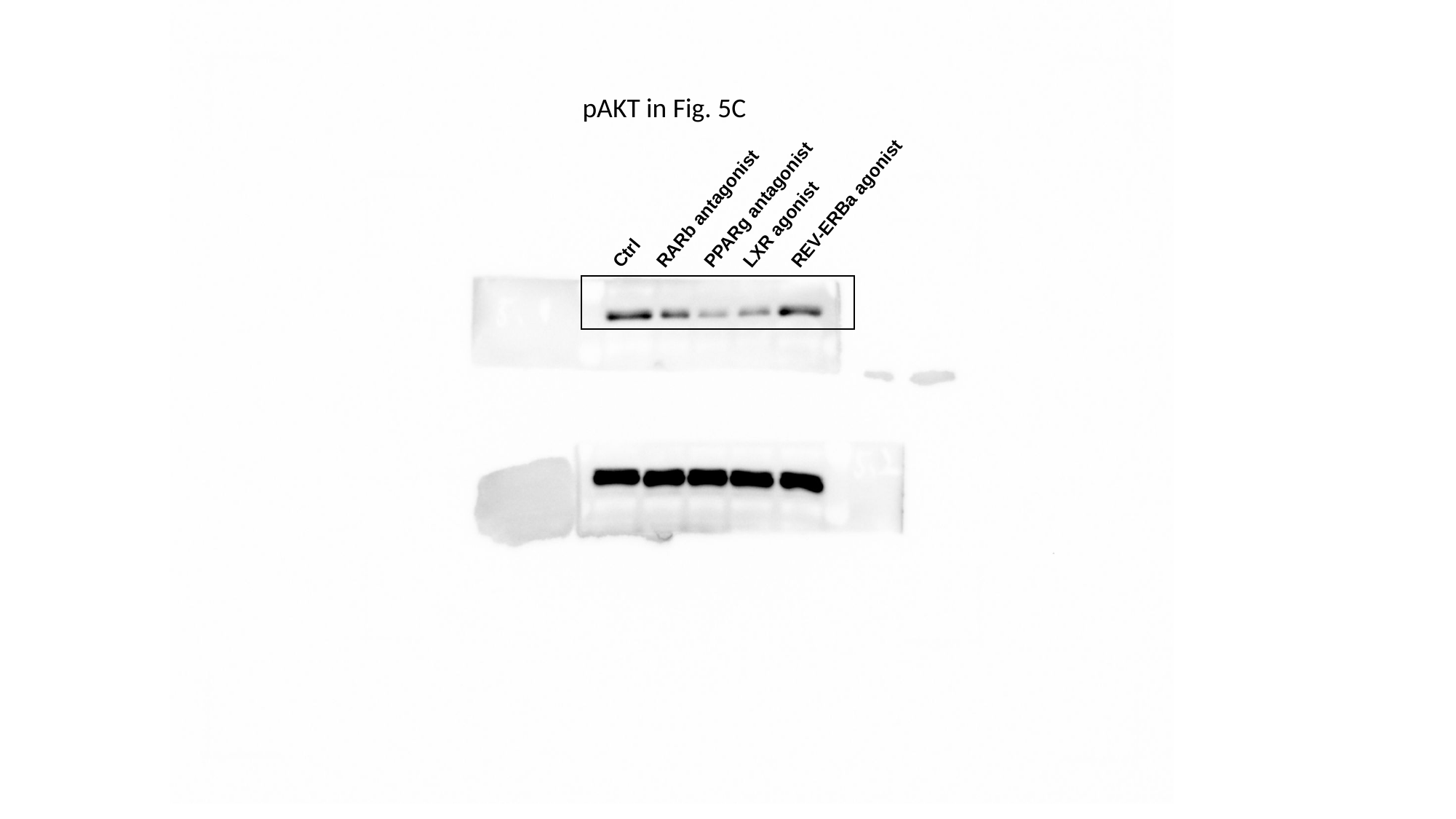

pAKT in Fig. 5C
RARb antagonist
PPARg antagonist
LXR agonist
REV-ERBa agonist
Ctrl

## Slide 14
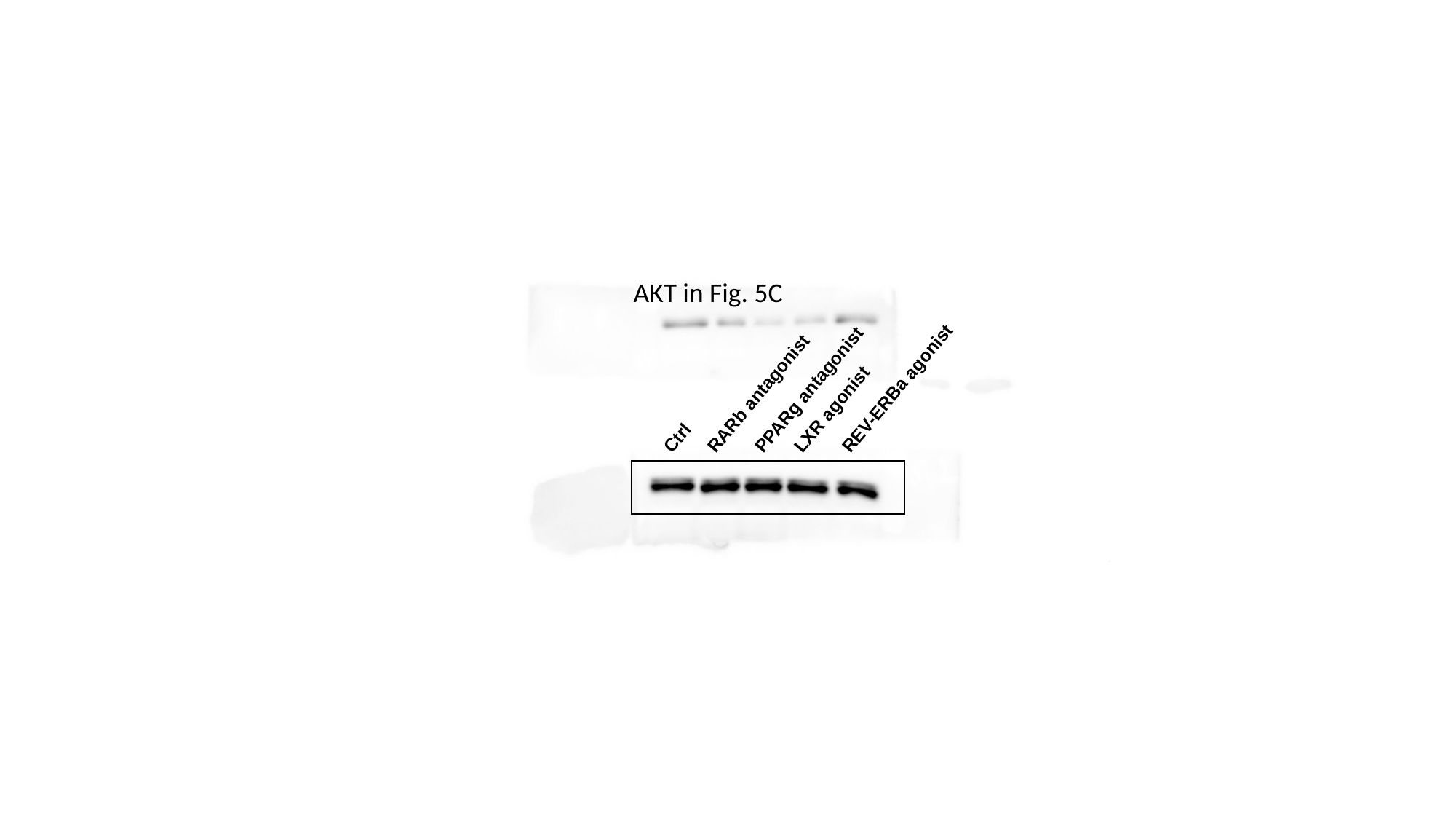

AKT in Fig. 5C
RARb antagonist
PPARg antagonist
LXR agonist
REV-ERBa agonist
Ctrl

## Slide 15
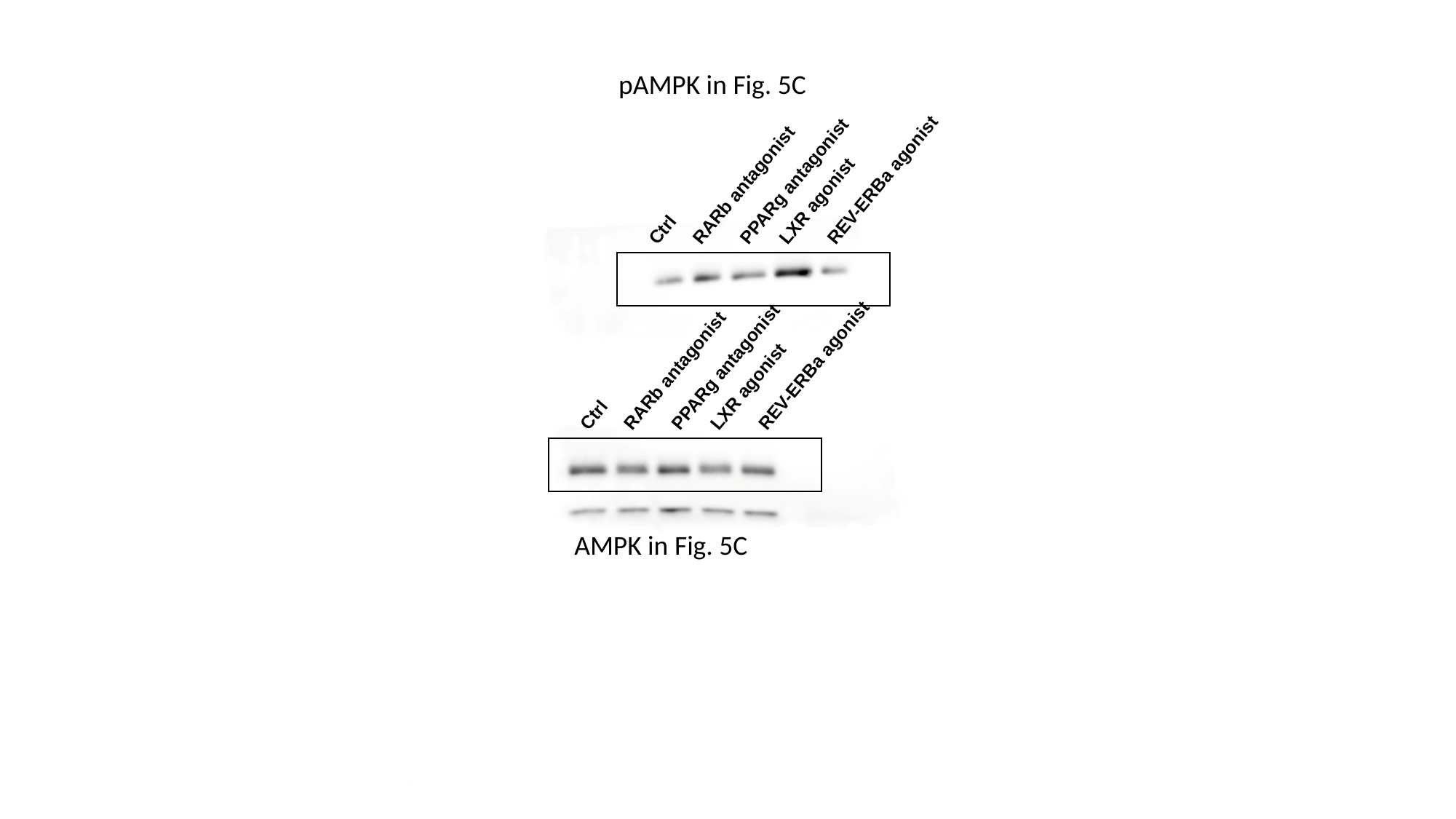

pAMPK in Fig. 5C
RARb antagonist
PPARg antagonist
LXR agonist
REV-ERBa agonist
Ctrl
RARb antagonist
PPARg antagonist
LXR agonist
REV-ERBa agonist
Ctrl
AMPK in Fig. 5C

## Slide 16
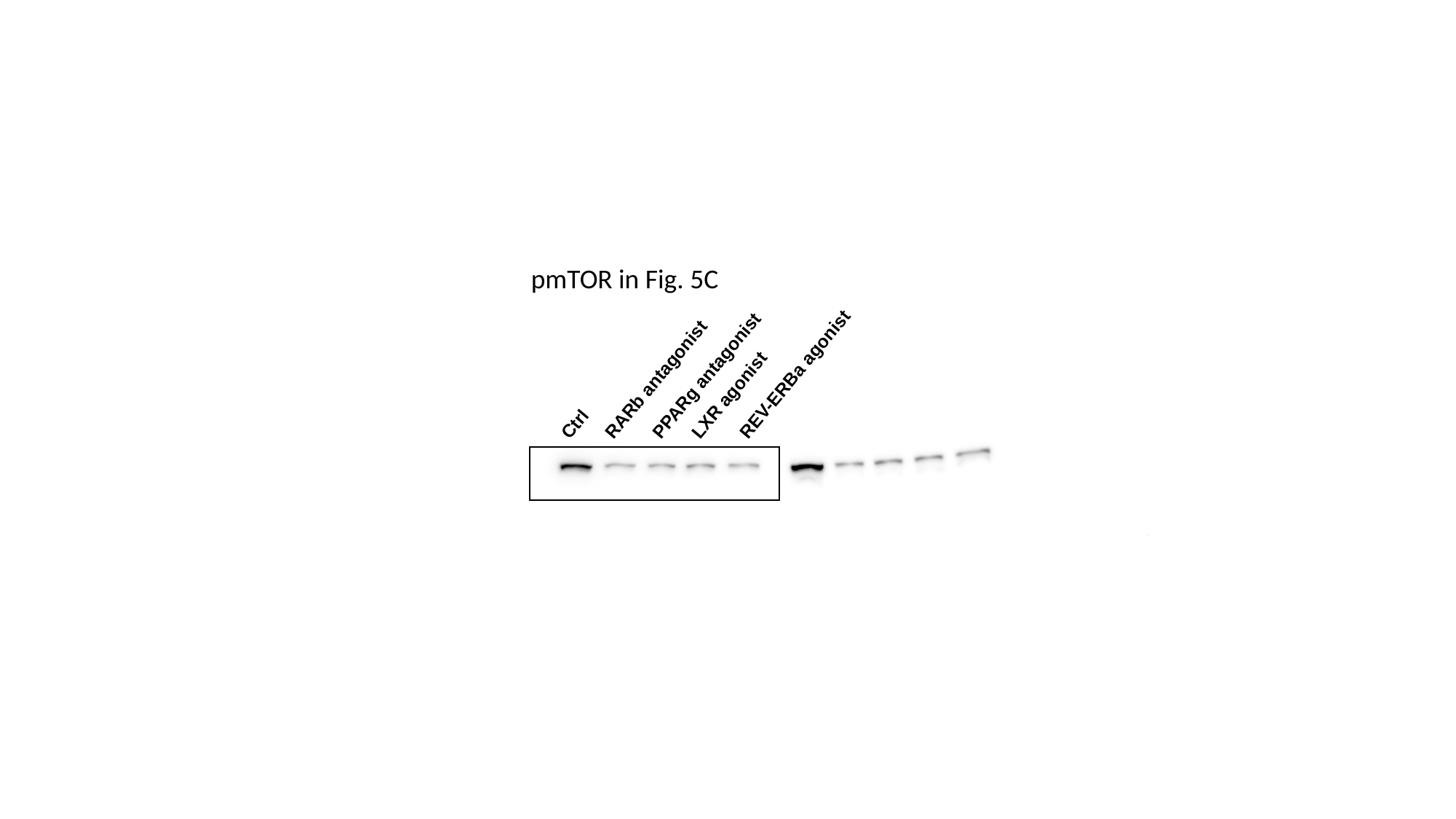

pmTOR in Fig. 5C
RARb antagonist
PPARg antagonist
LXR agonist
REV-ERBa agonist
Ctrl

## Slide 17
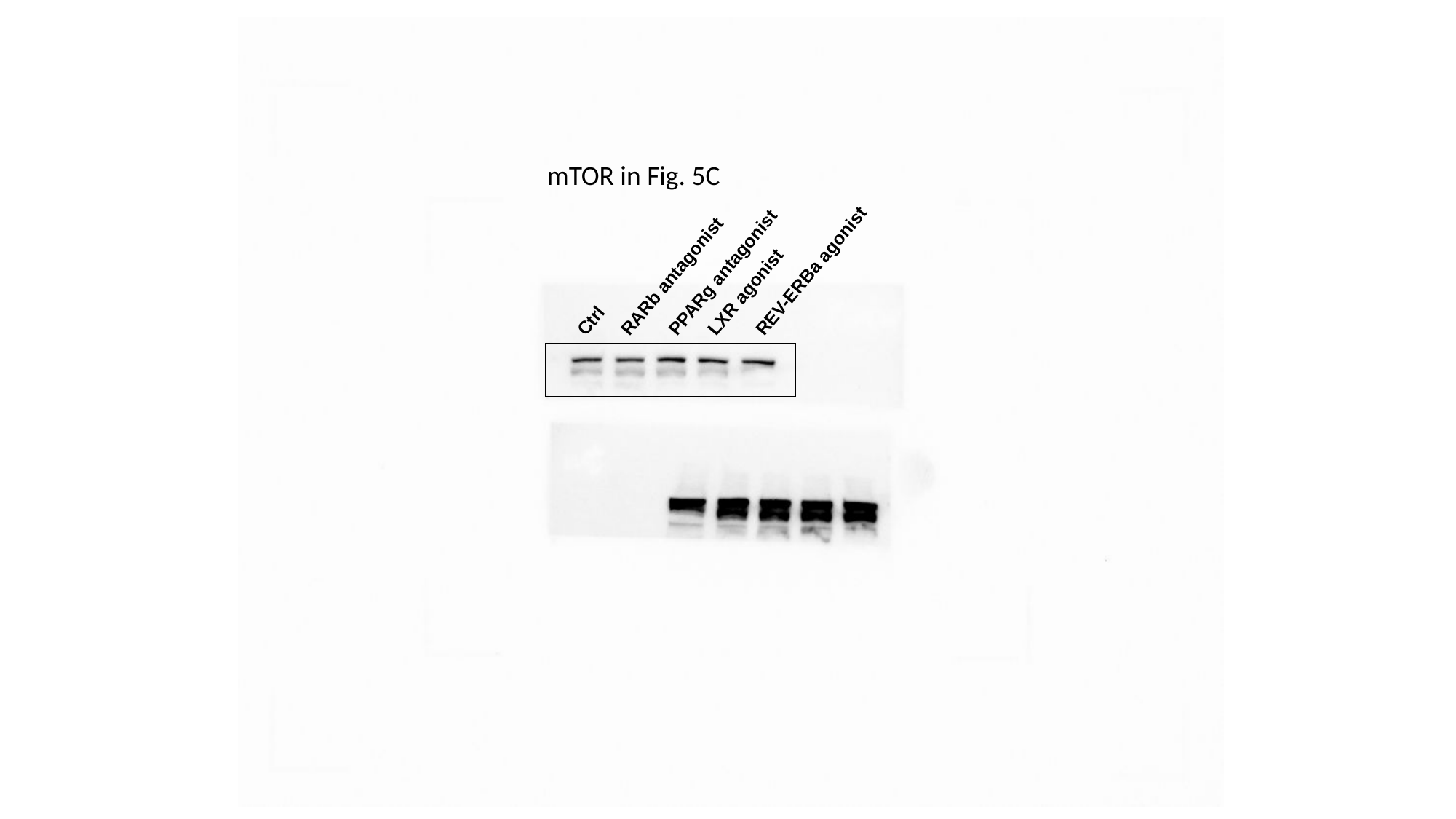

mTOR in Fig. 5C
RARb antagonist
PPARg antagonist
LXR agonist
REV-ERBa agonist
Ctrl

## Slide 18
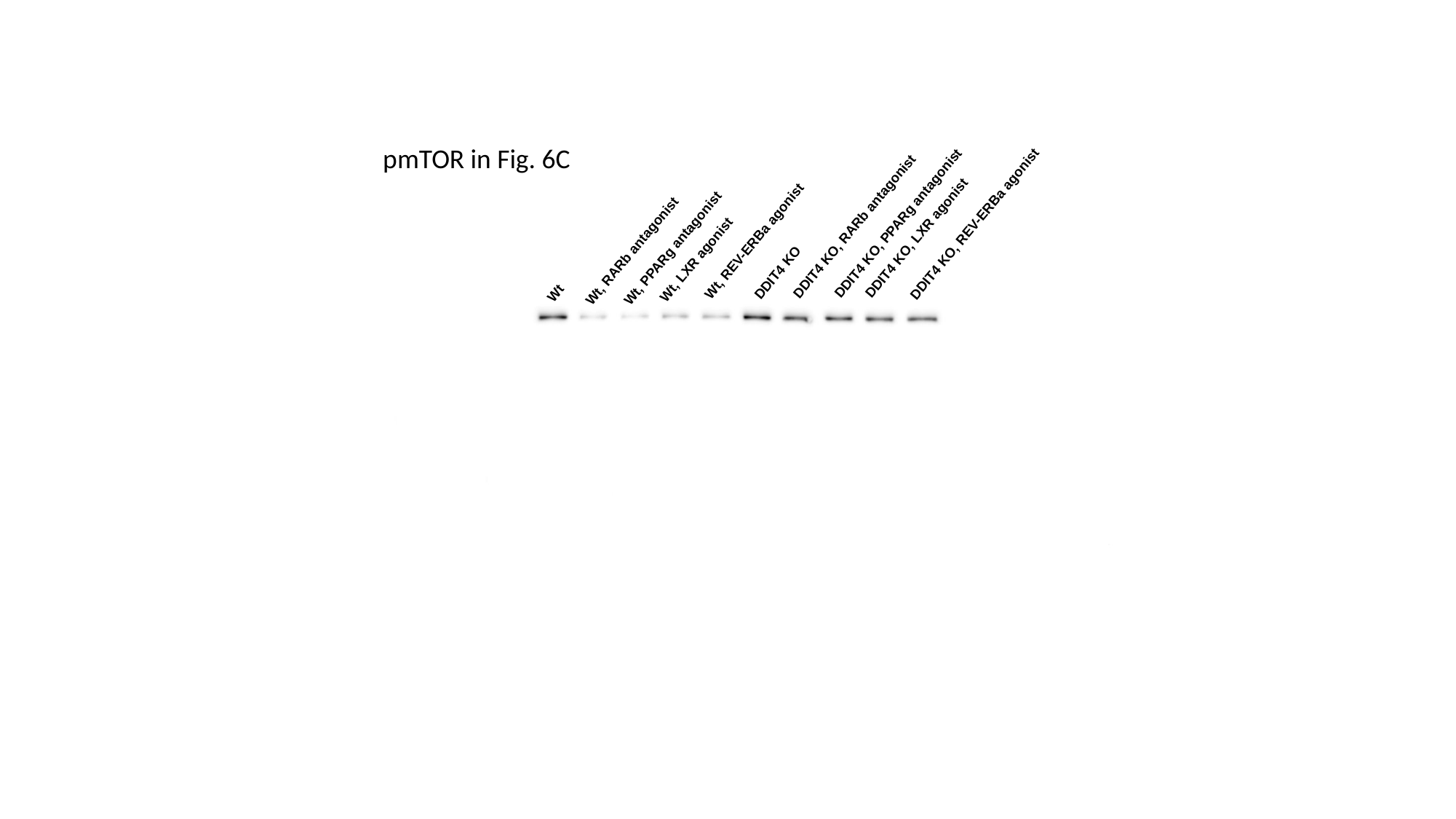

pmTOR in Fig. 6C
DDIT4 KO, PPARg antagonist
DDIT4 KO, RARb antagonist
DDIT4 KO, REV-ERBa agonist
DDIT4 KO, LXR agonist
Wt, PPARg antagonist
Wt, REV-ERBa agonist
Wt, RARb antagonist
Wt, LXR agonist
DDIT4 KO
Wt

## Slide 19
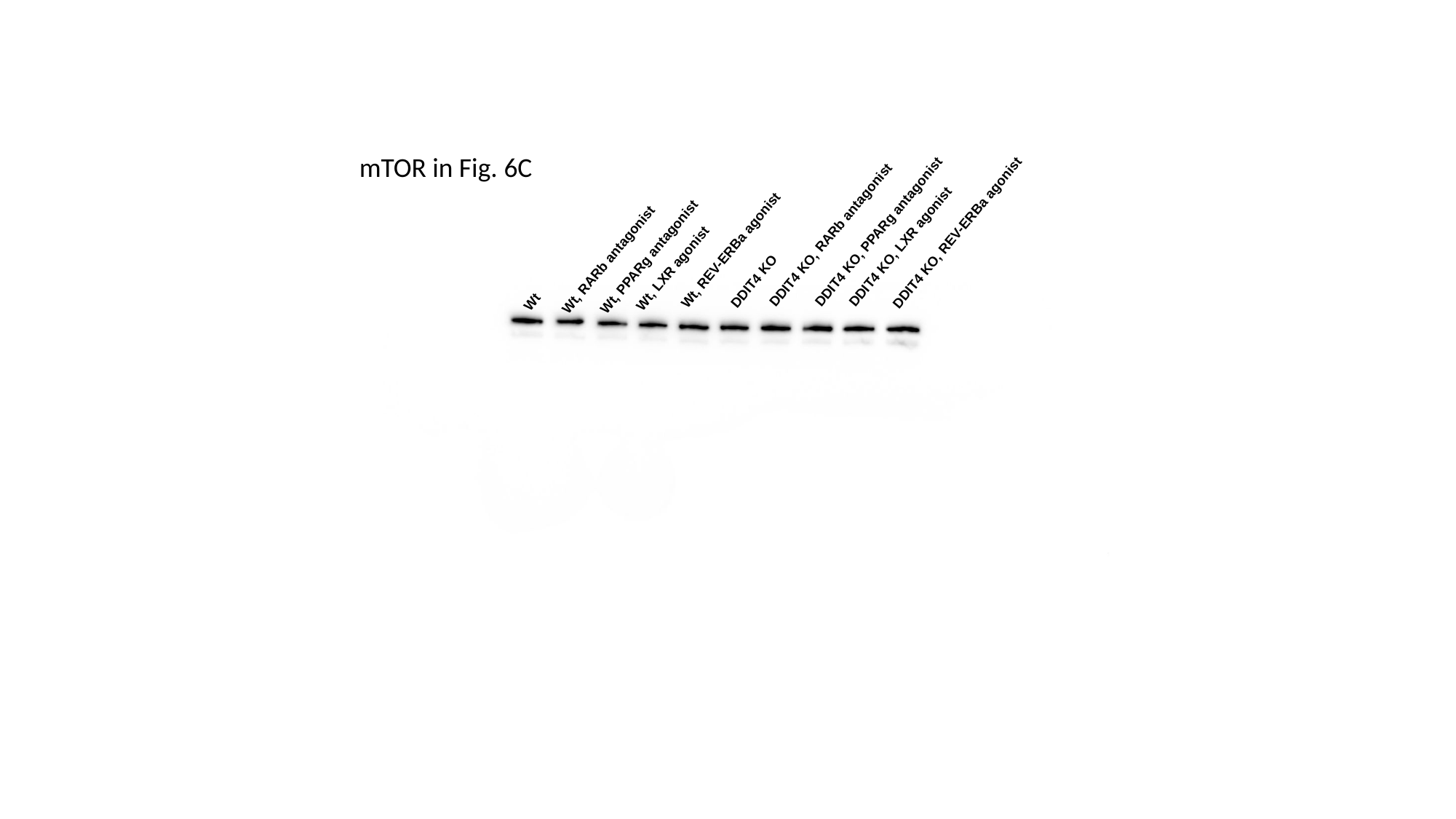

mTOR in Fig. 6C
DDIT4 KO, PPARg antagonist
DDIT4 KO, RARb antagonist
DDIT4 KO, REV-ERBa agonist
DDIT4 KO, LXR agonist
Wt, PPARg antagonist
Wt, REV-ERBa agonist
Wt, RARb antagonist
Wt, LXR agonist
DDIT4 KO
Wt

## Slide 20
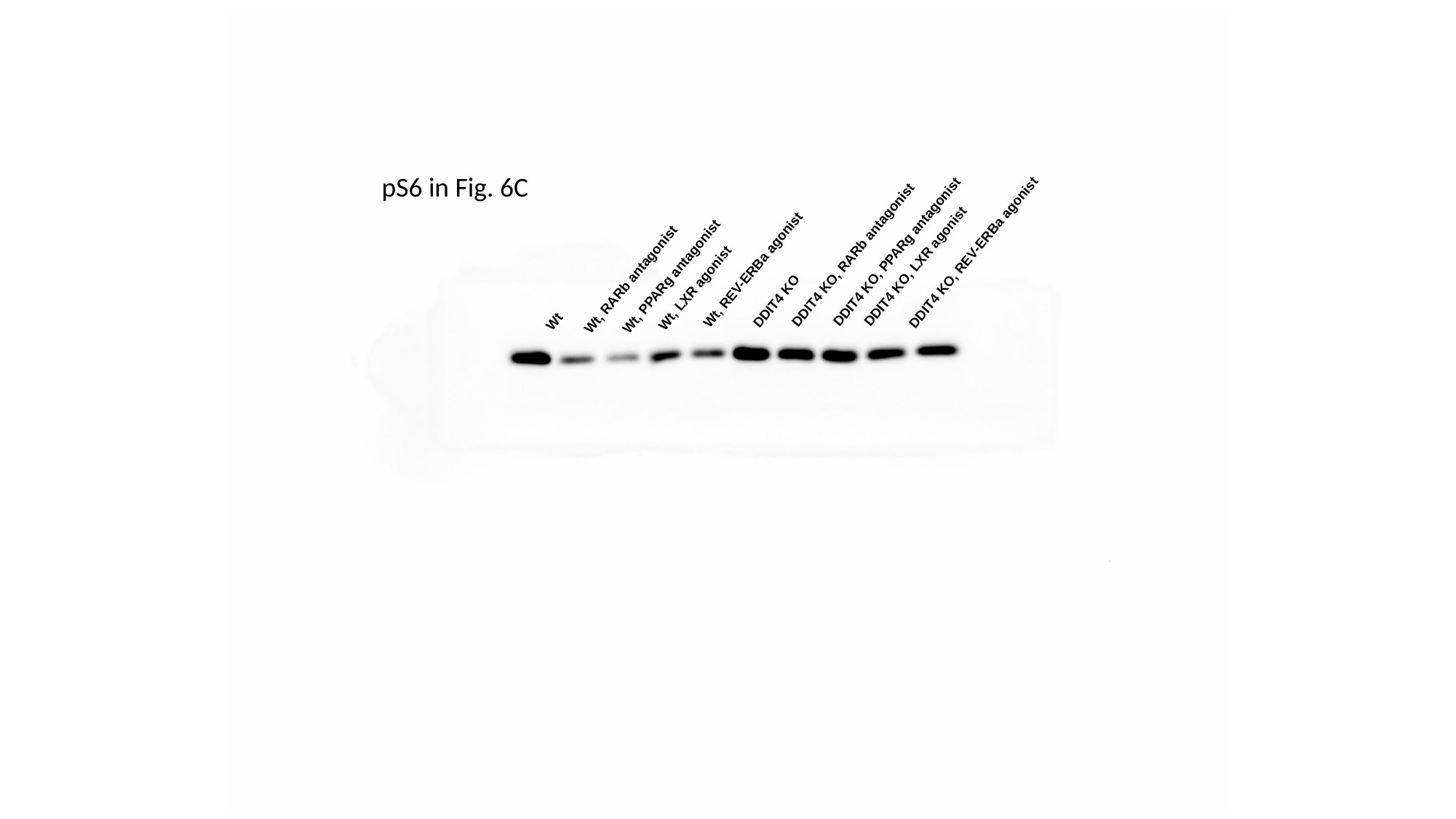

pS6 in Fig. 6C
DDIT4 KO, PPARg antagonist
DDIT4 KO, RARb antagonist
DDIT4 KO, REV-ERBa agonist
DDIT4 KO, LXR agonist
Wt, PPARg antagonist
Wt, REV-ERBa agonist
Wt, RARb antagonist
Wt, LXR agonist
DDIT4 KO
Wt

## Slide 21
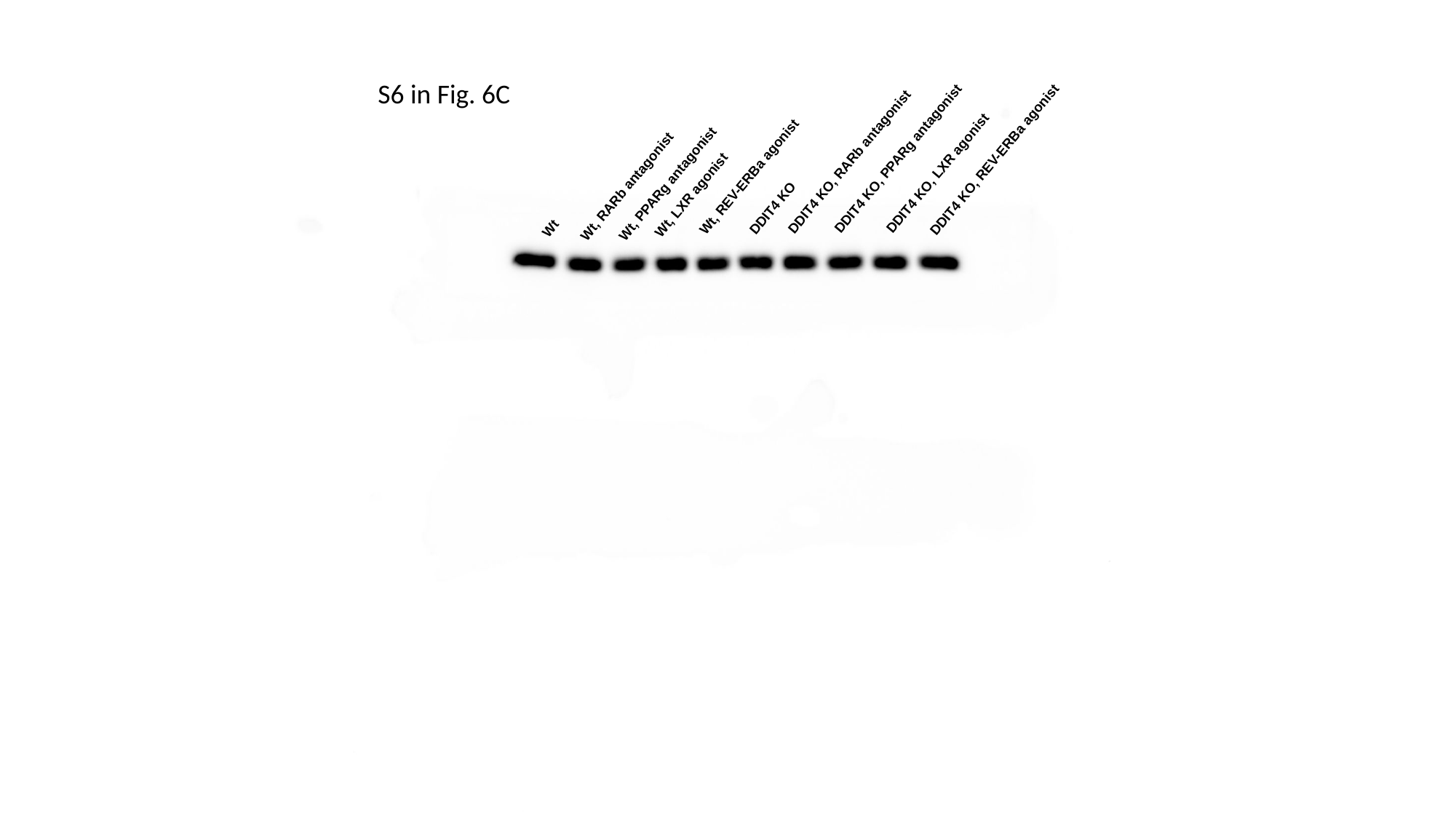

S6 in Fig. 6C
DDIT4 KO, PPARg antagonist
DDIT4 KO, RARb antagonist
DDIT4 KO, REV-ERBa agonist
DDIT4 KO, LXR agonist
Wt, PPARg antagonist
Wt, REV-ERBa agonist
Wt, RARb antagonist
Wt, LXR agonist
DDIT4 KO
Wt

## Slide 22
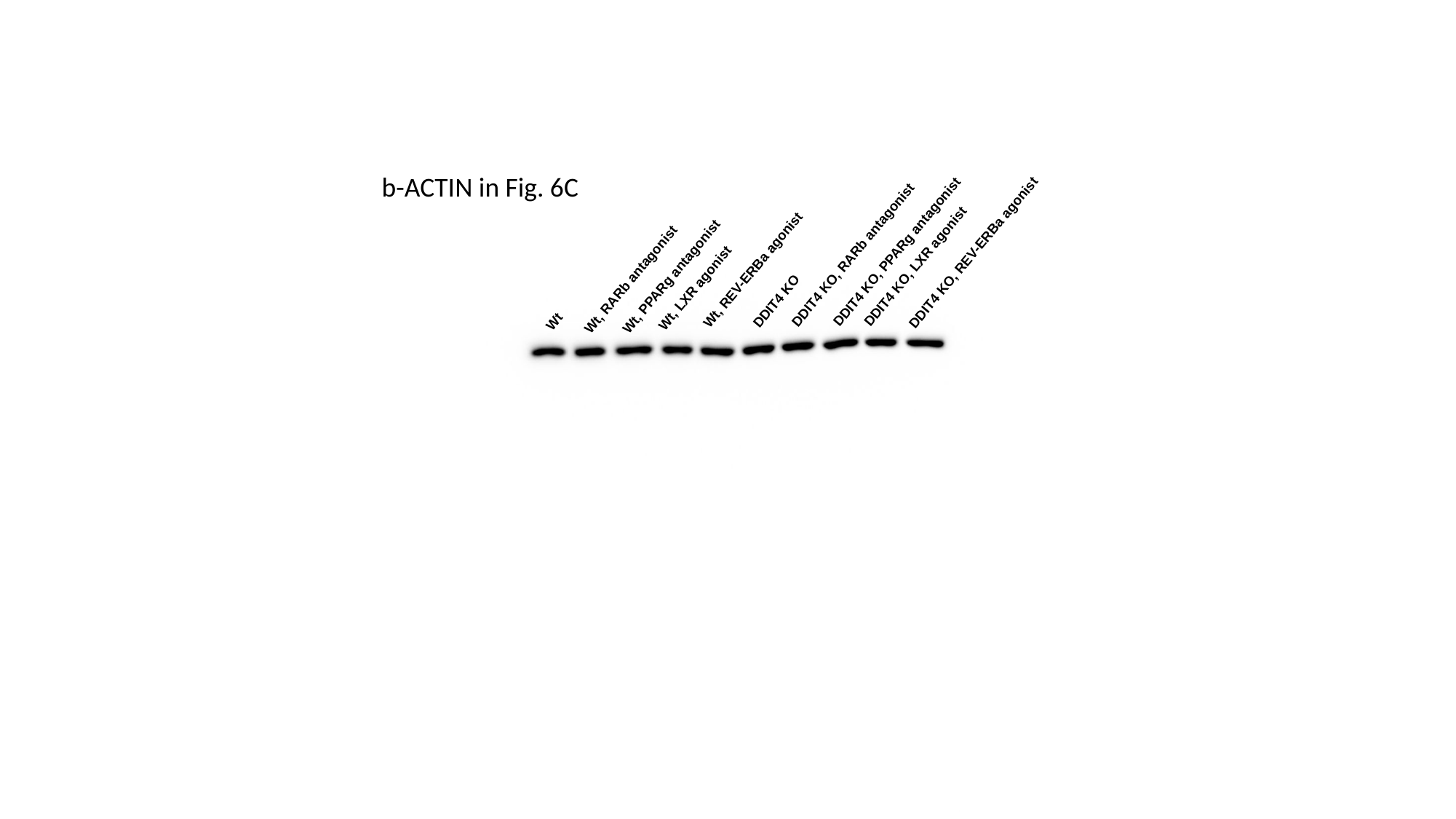

b-ACTIN in Fig. 6C
DDIT4 KO, PPARg antagonist
DDIT4 KO, RARb antagonist
DDIT4 KO, REV-ERBa agonist
DDIT4 KO, LXR agonist
Wt, PPARg antagonist
Wt, REV-ERBa agonist
Wt, RARb antagonist
Wt, LXR agonist
DDIT4 KO
Wt
