# Supplementary figures and images for: Nuclear receptor modulators inhibit osteosarcoma cell proliferation and tumour growth by regulating the mTOR signaling pathway
Source: Cell Death Dis. 2023 Jan 21;14(1):51. doi: 10.1038/s41419-022-05545-7 (PMC9867777; doi:10.1038/s41419-022-05545-7)

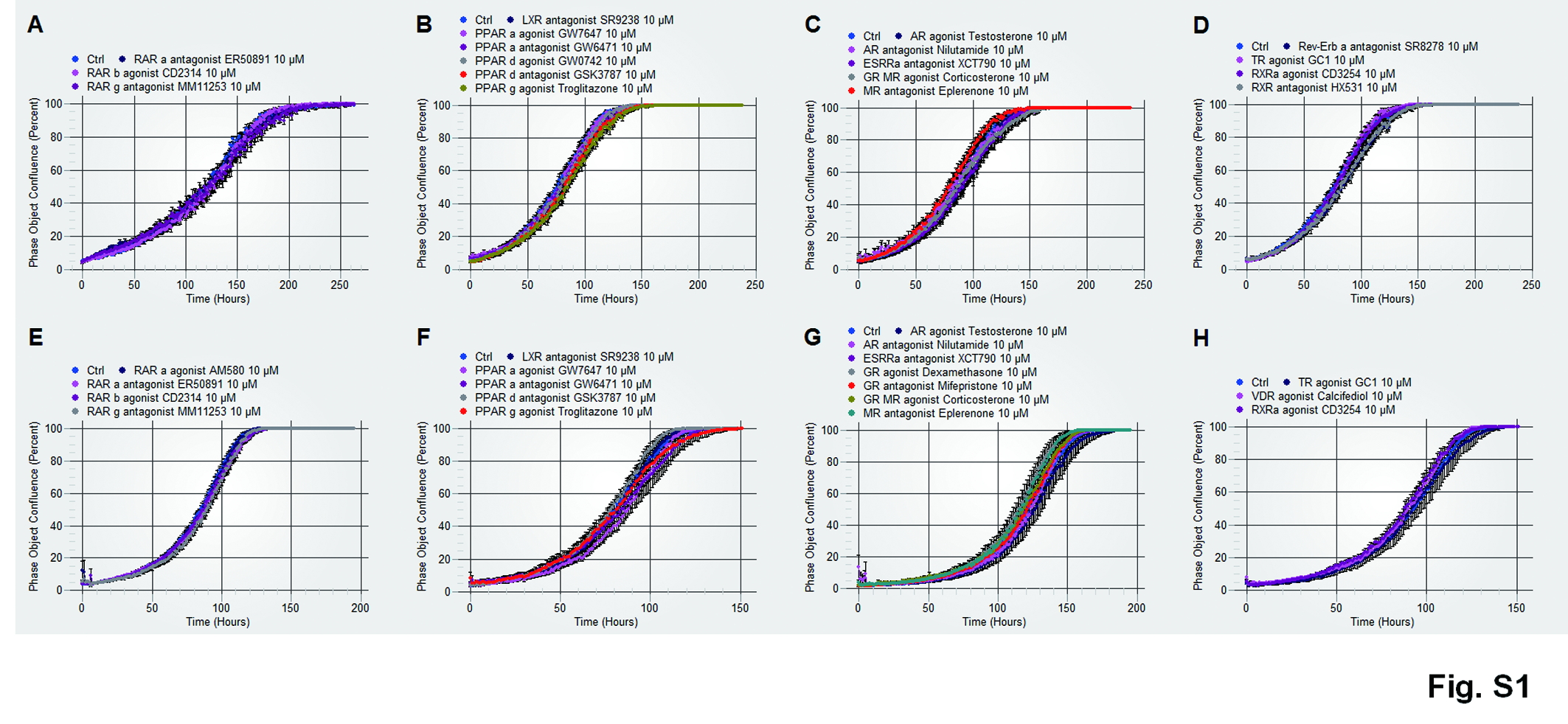

Supplement: Supplementary file 3 — Supplemental Figure 1 [file 41419_2022_5545_MOESM3_ESM.tif]

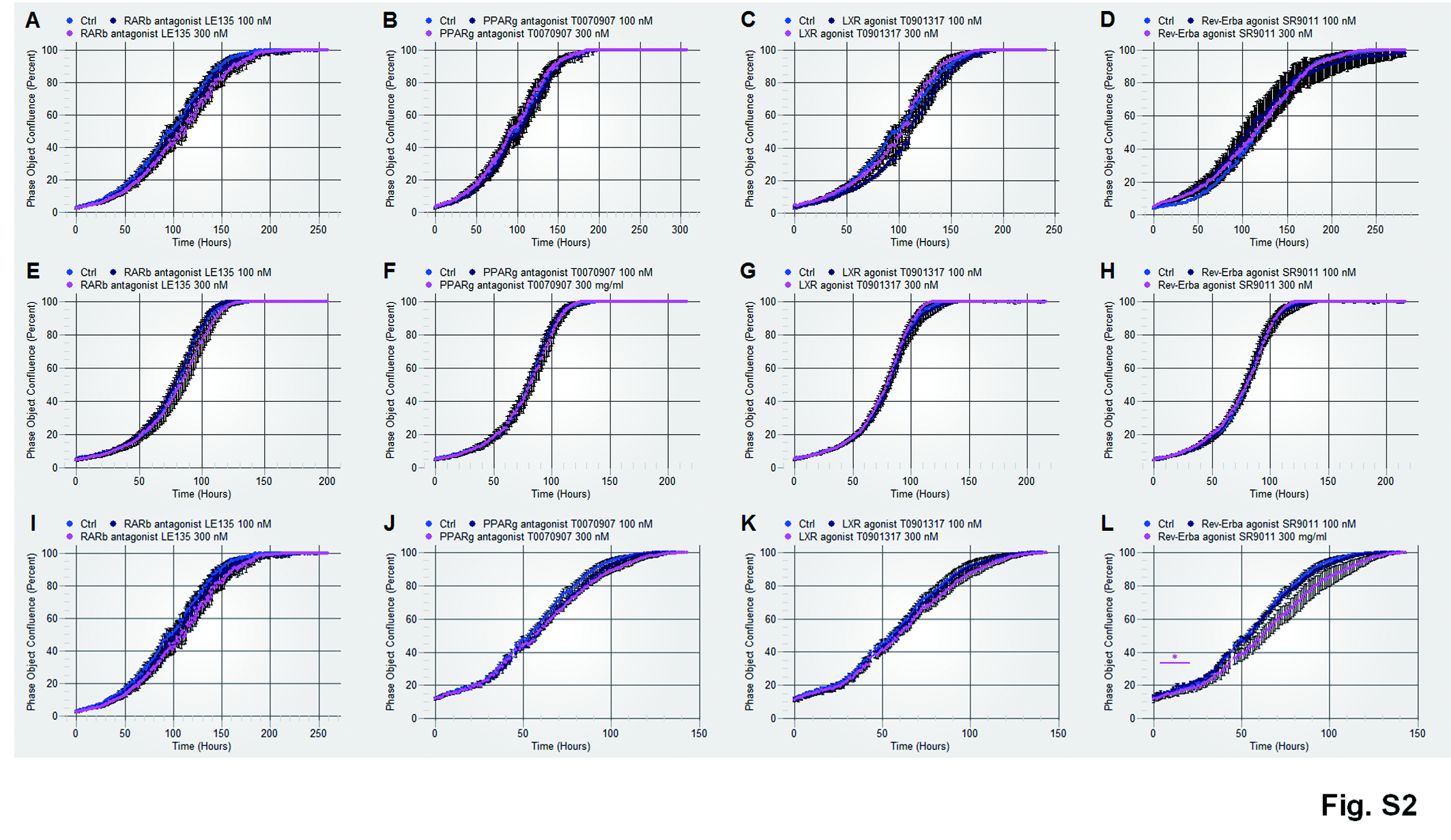

Supplement: Supplementary file 4 — Supplemental Figure 2 [file 41419_2022_5545_MOESM4_ESM.tif]

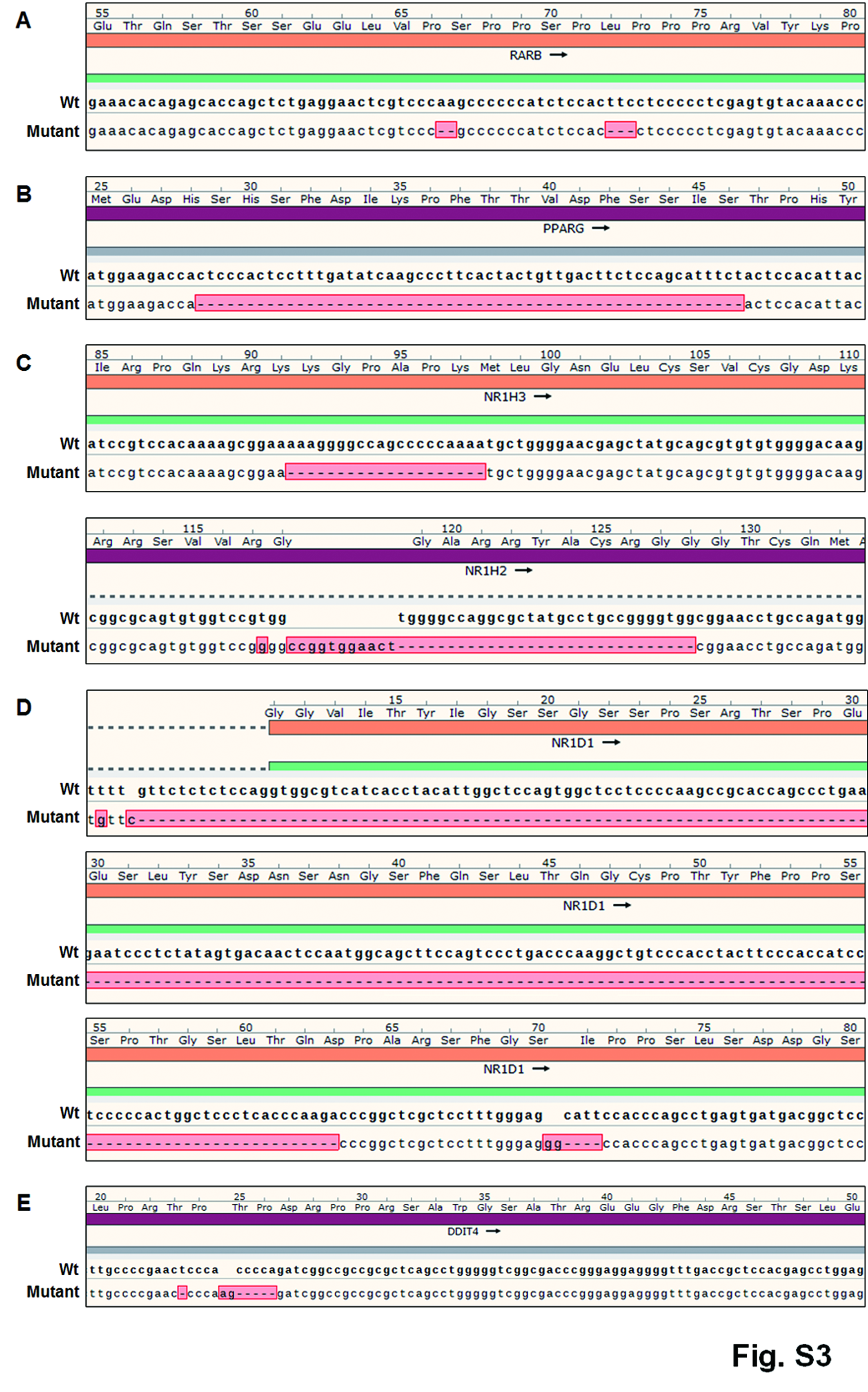

Supplement: Supplementary file 5 — Supplemental Figure 3 [file 41419_2022_5545_MOESM5_ESM.tif]

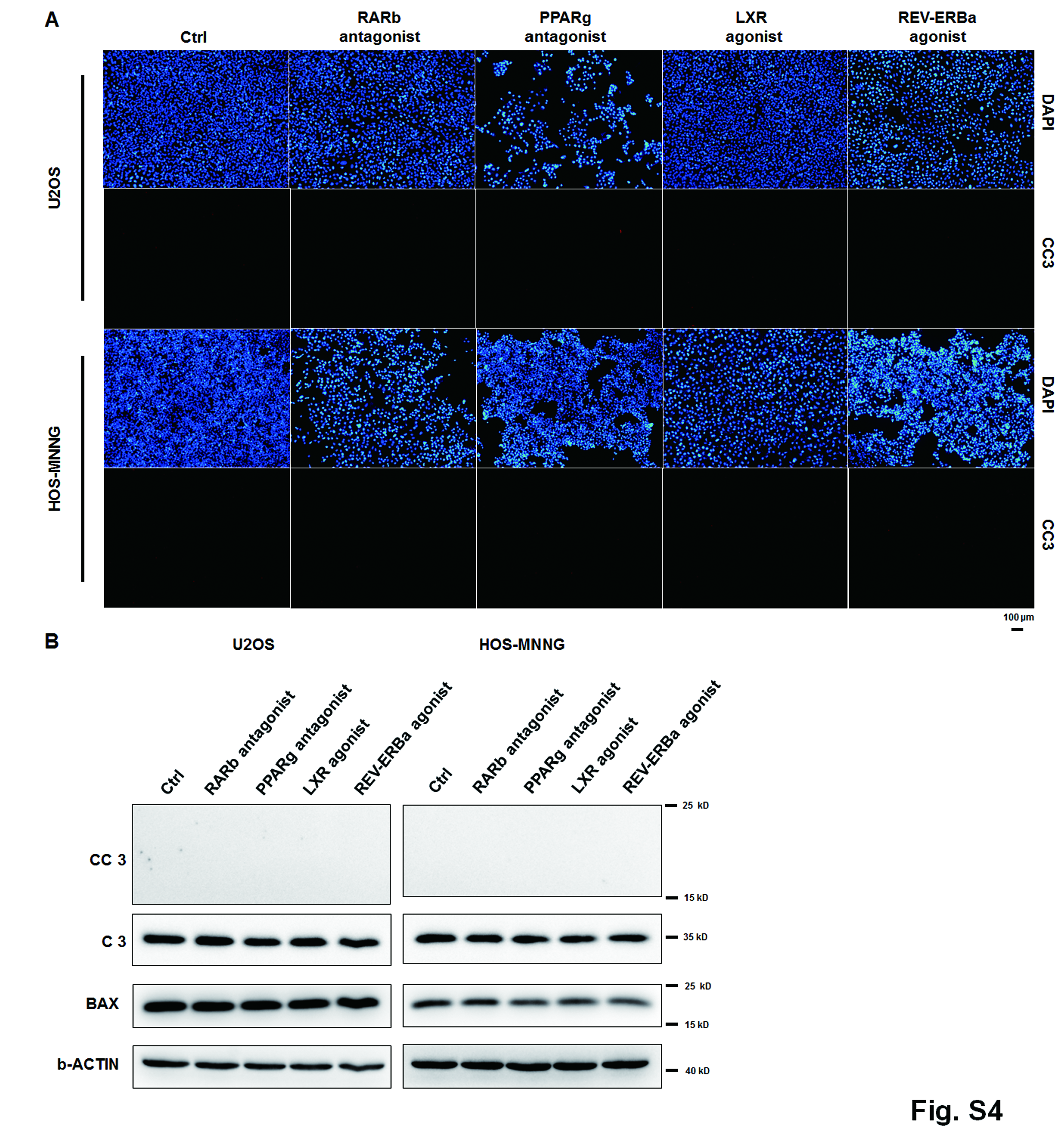

Supplement: Supplementary file 6 — Supplemental Figure 4 [file 41419_2022_5545_MOESM6_ESM.tif]

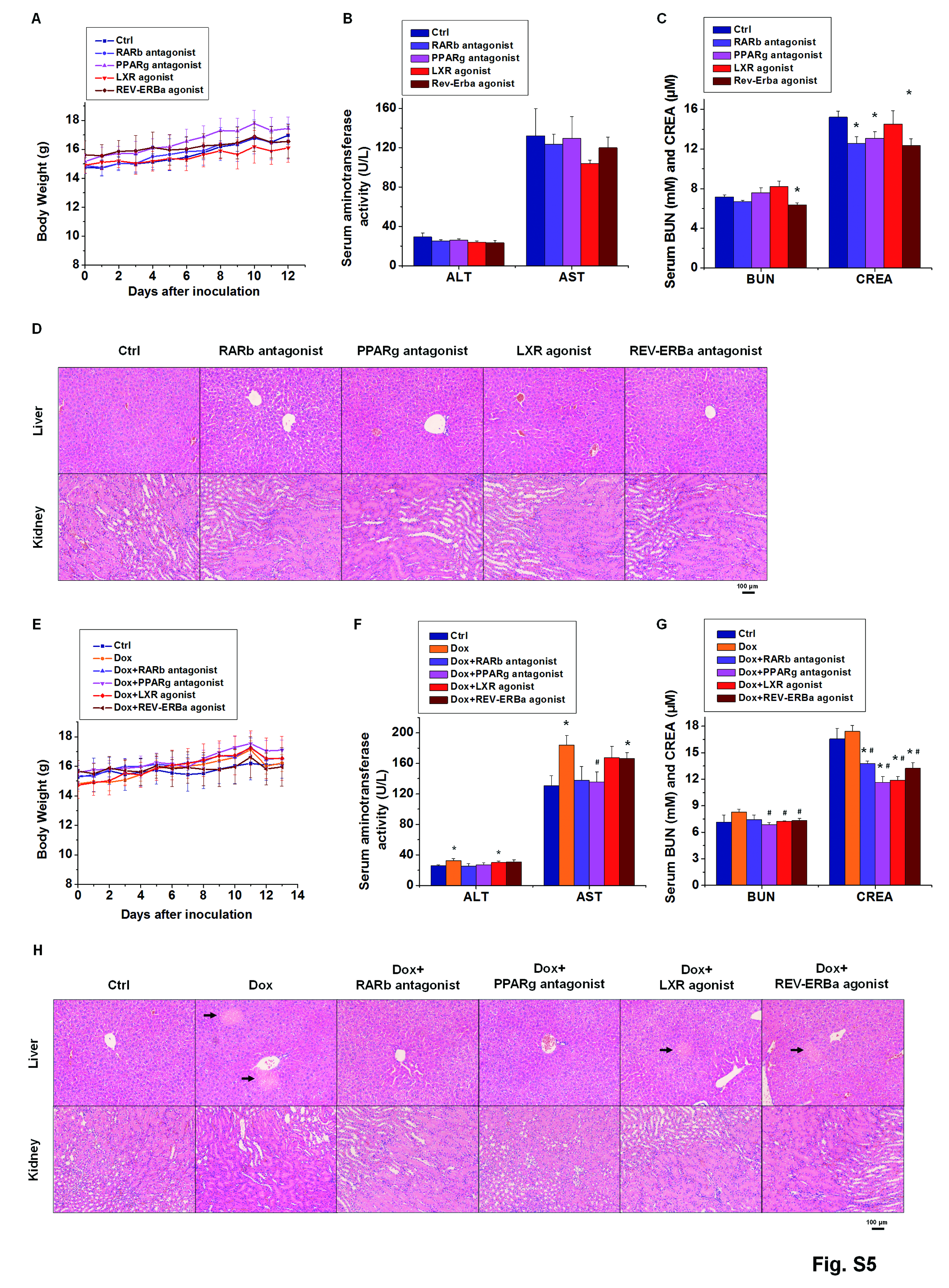

Supplement: Supplementary file 7 — Supplemental Figure 5 [file 41419_2022_5545_MOESM7_ESM.tif]
