# Supplementary material for: Nuclear receptor modulators inhibit osteosarcoma cell proliferation and tumour growth by regulating the mTOR signaling pathway
Source: Cell Death Dis. 2023 Jan 21;14(1):51. doi: 10.1038/s41419-022-05545-7 (PMC9867777; doi:10.1038/s41419-022-05545-7)
Supplement: Supplementary file 8 — Supplemental_Table_S1 [file 41419_2022_5545_MOESM8_ESM.docx]

**Table S1. The NR modulators used in the chemical screen and their corresponding NRs expressed in osteosarcoma cells.**

| **Nuclear receptor genes** | **FPKM in U2OS** | **FPKM in**  **HOS-MNNG** | **FPKM in Saos-2** | **Agonists** | **Antagonists** |
| --- | --- | --- | --- | --- | --- |
| RARa | 25.89±2.84 | 6.36±0.50 | 7.50±0.23 | AM 580, Retinoic acid | ER 50891, BMS 493 |
| RARb | 0.18±0.01 | 0.17±0.07 | 15.46±0.59 | CD 2314, Retinoic acid | LE 135, BMS 493 |
| RARg | 16.59±0.63 | 10.27±0.67 | 7.40±0.49 | CD 437, Retinoic acid | MM 11253, BMS 493 |
| PPARa | 3.11±0.72 | 0.47±0.03 | 3.23±0.31 | GW 7647 | GW 6471 |
| PPARd | 20.25±1.20 | 6.22±0.18 | 24.84±0.88 | GW 0742 | GSK 3787 |
| PPARg | 4.41±0.26 | 9.06±0.61 | 4.81±0.13 | Troglitazone | T 0070907 |
| LXRa | 2.62±0.05 | 1.22±0.24 | 4.71±0.28 | T 0901317 | SR 9238 |
| LXRb | 41.03±1.01 | 0.87±0.04 | 0.44±0.13 | T 0901317 | SR 9238 |
| THRa | 18.76±1.87 | 11.51±0.96 | 24.61±1.39 | GC 1 |  |
| THRb | 0.39±0.07 | 5.44±0.28 | 6.82±0.35 | GC 1 |  |
| VDR | 5.35±0.77 | 19.21±1.36 | 19.69±0.46 | Calcifediol |  |
| Rev-Erba | 6.61±0.59 | 8.63±0.16 | 9.53±0.71 | SR 9011 | SR 8278 |
| RXRa | 14.65±0.83 | 15.56±0.28 | 19.71±0.89 | CD 3254 | HX 531 |
| AR | 1.11±0.22 | 15.23±0.23 | 17.86±0.23 | Testosterone | Nilutamide |
| ESRRa | 15.45±0.28 | 0.88±0.08 | 3.98±0.29 |  | XCT 790 |
| GR | 15.35±1.39 | 22.41±0.37 | 17.26±0.52 | Dexamethasone, Corticosterone | Mifepristone |
| MR | 1.76±0.25 | 0.10±0.06 | 0.18±0.03 | Corticosterone | Eplerenone |

An FPKM value of 0.1 is set as the threshold for determining whether the gene is expressed or not.
